# Single-cell transcriptomics reveals the differential landscape of gonadal cells during MT-induced sex reversal in hermaphroditic protogynous orange-spotted grouper (*Epinephelus coioides*)

**DOI:** 10.1186/s12915-026-02695-4

**Published:** 2026-07-27

**Authors:** Xi Wu, Tong Wang, Leyi Chang, Yuhao Tao, Xiaotian Zhang, Yang Yang, Yanzhao Zhao, Leilei Zeng, Zining Meng, Xiaochun Liu

**Affiliations:** 1 State Key Laboratory of Biocontrol/Southern Marine Science and Engineering Guangdong Laboratory (Zhuhai), Key lab of Gungdong for improved variety reproduction of aquatic economic animals, School of Life Sciences, Sun Yat-Sen University, Guangzhou, 510275 China; 2 School of Marine Biology and Fisheries, Hainan Aquaculture Breeding Engineering Research Center, School of Breeding and Multiplication (Sanya Institute of Breeding and Multiplication), Hainan University, Haikou, 570228 China; 3 Key Laboratory of Tropical Marine Fish Germplasm Innovation and Utilization, Ministry of Agriculture and Rural Affairs, Hainan Engineering Research Center for Germplasm Innovation and Utilization, Hainan Chenhai Aquatic Co., Ltd, Sanya, 570000 China; 4 Lingshui Chenhai Seed Industry Co., Ltd., Lingshui, 572400 China; 5 China-ASEAN Belt and Road Joint Laboratory On Mariculture Technology, Guangzhou, 510275 China

**Keywords:** Sex reversal, Gonad, ScRNA-seq, Hermaphroditic, Orange-spotted grouper

## Abstract

**Background:**

Sex reversal is an intricate and fascinating process orchestrated by a finely organized network. The orange-spotted grouper (*Epinephelus coioides*) is a protogynous hermaphroditic fish, but the regulatory mechanism of its sex reversal is not yet well understood.

**Results:**

Here, single-cell transcriptome sequencing (scRNA-seq) of the gonads was performed during sex reversal induced by 17α-methyltestosterone (MT). Based on the scRNA-seq data, various gonadal cell types and subtypes, including eight types of germ cells and nine types of somatic cells, were characterized and identified. Moreover, a single-cell atlas of dynamic changes in gene expression during sex reversal in orange-spotted grouper was constructed. We outlined the precise trajectories of germ stem cells (GSCs) and male somatic cells, which revealed that new male germ cells were originated from a subcluster of GSCs with increased levels of spermatogenesis genes. Moreover, we revealed that *piwil1* and *dmrt1* were important for the male-oriented differentiation of GSCs. Additionally, oocytes exhibited abnormal and significant apoptosis and active proliferation during the early stage of sex reversal.

**Conclusions:**

Overall, these results address the questions of how male germ cells are generated and how female germ cells disappear during sex reversal in orange-spotted grouper. These findings profile a comprehensive dynamic map of gonadal cells during sex reversal, which can provide new insights into sex change in lower vertebrates.

**Supplementary Information:**

The online version contains supplementary material available at https://doi.org/10.1186/s12915-026-02695-4.

## Background

Sex determination is a biological process through which the sex of an organism is established. Once the sex is determined, the process by which the undifferentiated gonads begin to differentiate into testes or ovaries is known as sex differentiation. The mechanisms of sex determination in vertebrates can generally be divided into two types: genotypic sex determination (GSD) and environmental sex determination (ESD) [1]. In mammals and birds, sex determination is typically genotypic. These animals possess sex-determining genes, such as the *Sry* gene in mammals and the *dmrt1* gene in chickens [2, 3]. In contrast, reptiles, amphibians, and fish exhibit a more diverse range of sex determination mechanisms, which are influenced by various external environmental factors. Among them, the mechanisms of sex determination in fish are the most complex and diverse. Fish possess both GSD and ESD mechanisms. Some fish species, including *Oryzias latipes*, *Oncorhunchus mykiss*, and *Oreochromis niloticus*, have evolved the XX/XY male heterogametic system independently from mammals. Even within the XX/XY system, the genes that initiate male development vary [4–6]. Additionally, some fish species utilize the ZZ/ZW sex determination system, such as *Cynoglossus semilaevis* and *Oreochromisco aureus* [7, 8]. The coexistence of different sex determination systems has also been observed within the same species, for example, in zebrafish, guppies, and silver crucian carp. Moreover, the sex of many fish species can be influenced by various environmental factors; therefore, sex characteristics of the fish are not fixed [9]. The sex characteristics of all fish can be divided into two major categories: gonochorism and hermaphroditism [10]. There are three types of hermaphroditism: sequential protogyny (female first, then male), sequential protandry (male first, then female), and simultaneous hermaphroditism [11]. The molecular mechanisms underlying sex differentiation in hermaphroditic fish are still not well understood.

Sex reversal is a phenomenon in the life history of hermaphroditic fish in which spontaneous sex transformation occurs, essentially representing a process of sex differentiation. Currently, research on the molecular mechanisms of sex reversal in hermaphroditic fish is limited, with only a few reports on species such as the eel (*Monopterus albus*), grouper, black porgy, and Asian seabass (*Lates calcarifer*) [12]. Sun’s team reported that in hermaphroditic eels, both oogonial stem cells (OSCs) and spermatogonial stem cells (SSCs) coexist, which may explain the hermaphroditism phenomenon [13]. Additionally, Zhou’s team conducted scRNA-seq on the intersex gonads of eels and suggested that the bidirectional differentiation potential of eel GSCs is the cause of sex reversal [14]. The grouper is a protogynous hermaphroditic fish and an important marine economic fish species in China. When groupers reach sexual maturity for the first time, they are females, while after spawning for one or several years, some females undergo sex reversal and transform into males [15]. Owing to its unique reproductive mode, the grouper has long been a model organism for studying sex differentiation and sex reversal [16]. Many researchers have reported that the sex reversal process in grouper is regulated by multiple factors, including various hormones, enzymes involved in hormone synthesis, epigenetic regulatory factors, and social factors. For example, oestrogen, androgen, and cortisol can promote sex reversal in grouper [17–20]. Rate-limiting enzymes of steroid hormones, such as *hsd-b* [21], *star* [22], and *cyp19a1a* [23], play key roles in the sex reversal process. Moreover, genes such as *amh* [24, 25], *dmrt1* [26, 27], and *foxl* [27] are also involved in the sex reversal process. Furthermore, it has been reported that DNA methylation of the *cyp19a1a* promoter and changes in chromatin accessibility are involved in the sex reversal process [28, 29]. Additionally, the sex of grouper is influenced by social context, such as the disappearance of the dominant individual [30]. However, the molecular mechanisms underlying sex reversal in grouper are not yet fully understood. What are the origins of the male germ cells and gonadal somatic cells during sex reversal? How do the cells in the gonads change during sex reversal?

To answer these two questions, we induced sex reversal in orange-spotted grouper (*Epinephelus coioides*) by artificial implantation of androgen and conducted scRNA-seq on the gonads before and after sex reversal. In this study, by artificially inducing sex reversal in orange-spotted grouper, we compared the scRNA-seq data of the gonads before, during, and after sex reversal and from naturally male fish. We presented comprehensive transcriptional profiles of all the cells in the gonads, described the dynamic changes in all the cells during sex reversal, and analysed and validated the differentiation processes of GSCs and gonadal somatic cells during sex reversal. Based on our results, we speculated that Sertoli cells and Leydig cells were derived from granulosa cells and that all the male germ cells differentiated from GSCs. Our findings provide new insights into the molecular mechanisms of sex reversal in hermaphroditic fish and other vertebrates.

## Results

### Gonadal histology during MT-induced sex reversal in orange-spotted grouper

Orange-spotted groupers were implanted by MT to induce sex reversal for 9 weeks in the MT implantation group and blank rubber strip implantation group (*n* = 100). Initially, the gonads remained in a stage with abundant primary-growth oocytes (PGOs) and cortical-alveolus oocytes (PVOs) before MT implantation (Additional file 1: Fig. S1A). After MT implantation, the gonads of the fish in the control group also remained at the same stage in our previous experiments [28, 31]. The sex of the fish in the MT-implanted group changed from female to male over 6 weeks. Histologically, 1 week after MT implantation, the oocytes began to atrophy, and small numbers of spermatogonia (SG) and spermatocytes (SCs) appeared in the gonads. This stage was defined as the early phase of sex reversal (Week 1, Additional file 1: Fig. S1B). Three weeks after implantation, the gonads were characterized by numerous SGs, SCs, and a limited number of oocytes (Week 3, Additional file 1: Fig. S1C). Six weeks after MT implantation, the gonads entered the late stage of sex reversion, with the majority of the male germ cells present, similar to natural testes (Week 6, Additional file 1: Fig. S1D). However, at 7 weeks after MT implantation, compared with the gonads at 6 weeks, 50% of the gonads possessed abundant oocytes and fewer male germ cells (Week 7, Additional file 1: Fig. S1E). Finally, at 9 weeks after MT implantation, all gonads reverted to ovary-like gonads with many oocytes and few male germ cells (Week 9, Additional file 1: Fig. S1F). We also detected the expression profiles of marker genes related to pluripotency (*dnd*, *esg1*, *nanog*, *pou5f1*), spermatogenesis (*dmrt1*, *npm1*), and oogenesis (*h1foo*, *slbp2*, *ybx2a*, *zar1*) during sex reversal. The results revealed that the expression levels of pluripotent stem cell marker genes were generally lowest at week 6 (Additional file 1: Fig. S1G–J). The expression levels of male germ cell marker genes gradually increased during the first 6 weeks, peaked at the 6th week, and then decreased (Additional file 1: Fig. S1K, L). In contrast, the expression levels of female germ cell marker genes tended to decrease gradually during the first 6 weeks but then gradually increased after the 6th week, reaching levels consistent with those in normal female gonads (Additional file 1: Fig. S1M–P). These results are consistent with the previously observed gonadal morphological changes.

### ScRNA-seq overviews of the gonadal cells during sex reversal in orange-spotted grouper

**Fig. 1:**
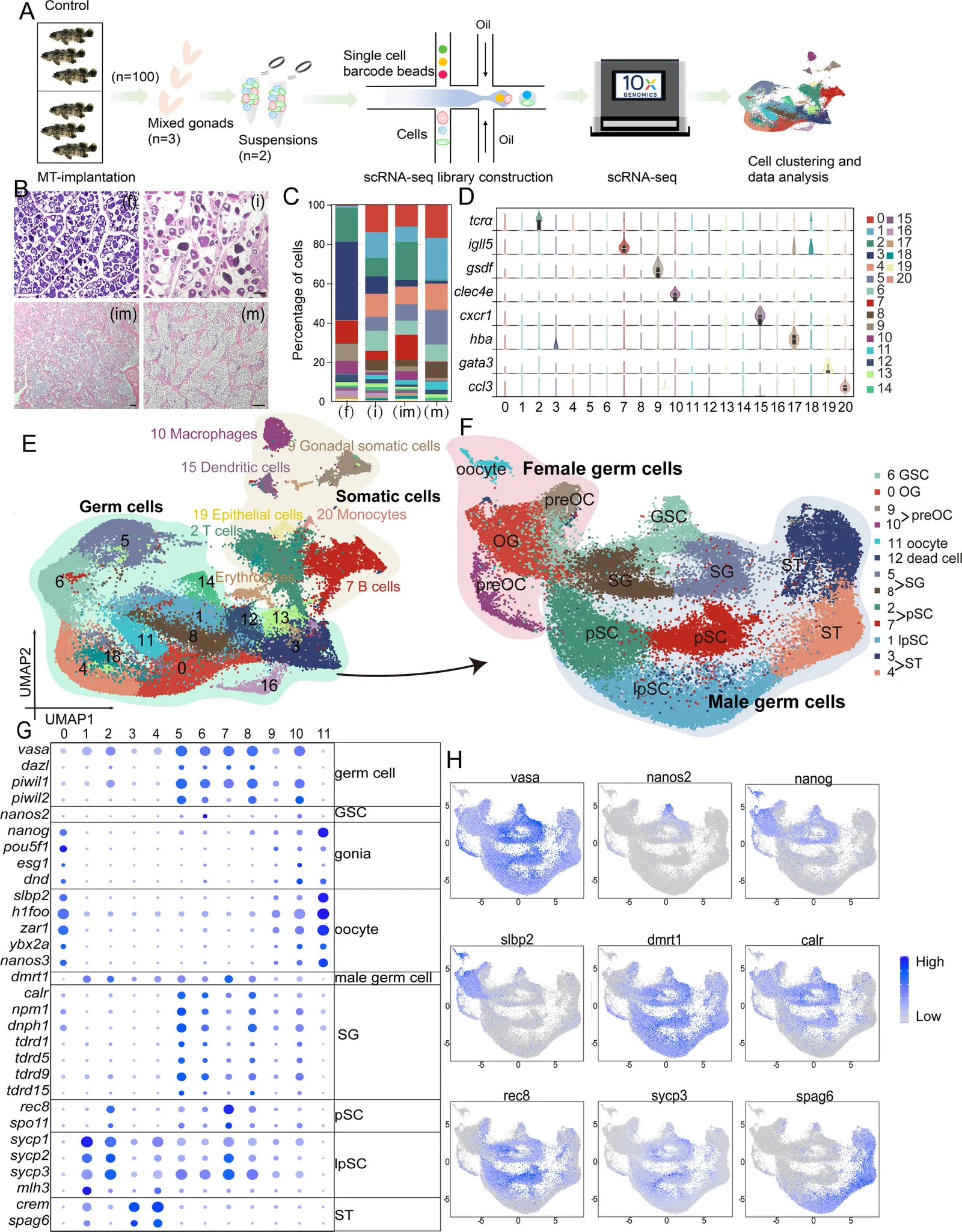
Experiment design and the overview of scRNA-seq data. **A** Overview of the whole experiment. The fish were divided into two groups, control group and MT implantation group. All the experiment lasted 9 weeks. The fish from two groups were dissected from cloacal aperture, and the gonad was taken out. After microscopy view of the gonad, four stages of gonads (a, ovary without implantation; b, intermediate stage of sex reversal in MT implantation group; c, late stage of sex reversal in MT implantation group; d, natural testis) were dissociated into single-cell suspension for sequencing (*n* = 2) and analysis. **B** Gonadal histological morphology of the orange-spotted groupers by H&E staining at four stages above. (f), natural female; (i), the intermediate stage of sex reversal; (im), MT induced male; (m), natural male. Scale bar = 50 μm. **C** Histogram of the percentage of cells in four different gonads (f, i, im, and m). **D** Violin plots of marker genes in somatic cells (cluster 2, 7, 9, 10, 15, 17, 19, 20). **E** UMAP plot of total cells clusters among eight samples. Colours marked the 21 distinct clusters were identified by unsupervised clustering and characterized with differential gene expression and gene set enrichment analyses. Each dot represents a cell. Clusters in yellow circle were defined by somatic cells, and clusters in green circle were defined by germ cells. **F** UMAP plot of germ cell clusters from panel **E**. Clusters in red circle were defined by female germ cells, and clusters in blue circle were defined by male germ cells. **G** Dot-plot showing the relative expression of select genes in germ cell clusters. Some clusters, such as cluster3 and cluster4, cluster2 and cluster7, cluster5 and cluster8, and cluster9 and cluster10 possess similar marker genes; therefore, they are classified as the same cell type. **H** UMAP plots of 9 marker genes in all germ cell clusters. The bluer dots represent higher expression. GSC, germ stem cell; OG, oogonia; preOC, pre-oocyte; SG, spermatogonia; pSC, primary spermatocyte; lpSC, late primary spermatocyte; ST, spermatotid

To determine the changes in all the gonadal cells during sex reversal, we obtained eight gonadal samples from orange-spotted groupers at four different developmental stages [(f), natural female; (i), intermediate stage of sex reversal; (im), MT induced male; (m), natural male; *n* = 2]. First, we quickly determined the developmental stage of each individual’s gonad using a simple tissue squashing technique. The target gonadal samples were digested into cell suspensions derived from a mixture of gonads from three individuals (Fig. 1A). The prepared cell suspensions were subjected to scRNA-seq (10 × Genomics). The morphology of the single-cell suspensions and the gonadal tissues were subsequently characterized (Additional file 1: Fig. S2A, Fig. 1B). With respect to the scRNA-seq data, we obtained a total of 61,011 high-quality cells after stringent cell filtration, from which an average of 4583 transcripts (counted as unique molecular identifiers, UMIs) were detected, with 1306 genes in each cell (Additional file 1: Fig. S2B–C, Additional file 2: Table S1). Seurat was used to identify cell clusters, and uniform manifold approximation and projection (UMAP) was used for visualization. Ultimately, a total of 21 cell clusters were identified with many marker genes whose expression levels differed across the four gonadal stages (Fig. 1C, D).

Given the large number of cell clusters, we first chose to divide all the cell clusters into two groups: germ cells and somatic cells. Two groups of validated germ cell marker genes, namely, *vasa*, *dazl*, *piwil1*, and *piwil2* [32–34], were identified. As shown in Additional file 1: Fig. S3, the cell clusters with high expression levels of these four genes were concentrated in the lower-left corner of the UMAP plot, comprising a total of 13 clusters (Clusters 0, 1, 3, 4, 5, 6, 8, 11, 12, 13, 14, 16, and 18). Therefore, we defined these clusters as germ cell clusters, accounting for 73% of all the gonadal cells (Additional file 2: Table S2). The remaining clusters were defined as somatic cells, which were primarily concentrated in the upper-right corner of the UMAP plot, including Clusters 2, 7, 9, 10, 15, 17, 19, and 20, accounting for 27% of all the gonadal cells (Additional file 2: Table S2). Based on known marker genes and differentially expressed genes (DEGs), we classified the somatic cells into eight distinct cell types: (1) T cells (Cluster 2), (2) B cells (Cluster 7), (3) gonadal somatic cells (Cluster 9), (4) macrophages (Cluster 10), (5) dendritic cells (Cluster 15), (6) erythrocytes (Cluster 17), (7) epithelial cells (Cluster 19), and (8) monocytes (Cluster 20). Among them, T cells and B cells, marked by *tcra* (T-cell receptor alpha chain) [35], *cd28* (T-cell-specific surface glycoprotein CD28) [36], and *igll5* (immunoglobulin lambda-like polypeptide 5) [37] (Additional file 1: Fig. S3A–C), were the most abundant cell types, comprising 11,572 cells. A cluster of 1747 cells was identified as macrophages marked by *clec4e* (C-type lectin domain family 10 member A isoform X1) [38] and *mrc1*(macrophage mannose receptor 1-like) [39] (Additional file 1: Fig. S3D, E). Moreover, a cluster of 1768 cells was marked by the gonadal somatic cell markers *gsdf* [40, 41], *amh* [24, 42], and *gata6* [43] (Cluster 9, Fig. 1D; Additional file 1: Fig. S3F–H). Other clusters included dendritic cells (chemokine XC receptor 1-like, *xcr1*) [44], erythrocytes (haemoglobin alpha chain, *hba*; haemoglobin beta chain, *hba1*, and haemoglobin subunit alpha-1-like, *hbb2*), epithelial cells (*gata3* [45]; smooth muscle cell-specific protein SM22 alpha, *sm22α*) [43], and monocytes (C–C motif chemokine 3, *ccl3*) [46] (Fig. 1D–E, Additional file 1: Fig. S3I–N).

After the somatic cells from the gonads were filtered out, we performed a new dimensional reduction analysis on the integrated germ cells from the gonads. After the dead cells (Cluster 12) were filtered, the results revealed that all the germ cells reclustered into 12 cell clusters, which were defined as 8 cell types, including oogonia (OG, Cluster 0), late primary spermatocytes (lpSCs, Cluster 1), primary spermatocytes (pSCs, Clusters 2 and 7), spermatids (STs, Clusters 3 and 4), spermatogonia (SG, Clusters 5 and 8), GSCs (germ stem cells, Cluster 6), preoocytes (preOCs, Clusters 9 and 10), and oocytes (OCs, Cluster 11) (Fig. 1F). The UMAP plot revealed that all cells could be divided into two major groups, which were classified as female and male germ cells based on the expression of marker genes (Fig. 1F). All the germ cells expressed the four genes*—vasa*, *dazl*, *piwil1*, and *piwil2*—with relatively low expression levels in the oocytes, which is consistent with our previous results [32, 43]. Cluster 6 was defined as GSCs due to the expression of the known GSC marker *nanos2* [47]. Female germ cells (Clusters 0, 9, 10, and 11) expressed many maternal factors, *slbp2*, *h1foo*, *zar1*, *ybx2a*, and *nanos3* [31, 43, 47], and pluripotent genes, including *nanog*, *pou5f1*, *esg1*, and *dnd* [33, 48, 49]. Therefore, oocytes were characterized by the most abundant expression of the abovementioned maternal factors; OG were characterized by the most abundant expression of the abovementioned pluripotency genes, and preOCs expressed both types of genes. In addition, male germ cells (Clusters 1, 2, 3, 4, 5, 7, and 8) were marked by *dmrt1* [26]. Among them, SG (Clusters 5 and 8) expressed many identified markers, including *calr*, *npm1*, and *dnph1*, and several *tdrd* family genes, such as *tdrd1*, *tdrd5*, *tdrd9*, and *tdrd15* [50]. PSCs (Clusters 2 and 7) were labeled with *rec8*, *spo11*, *sycp2*, and *sycp3*, lpSCs (Cluster 1) were tabbed by *sycp1* and *mlh3*, and STs (Clusters 3 and 4) were stamped by *crem* and *spag6* [50] (Fig. 1G–H). After defining all the cell clusters, we found that the UMAP results of all the germ cells essentially outlined the primary differentiation trajectory of the cells during the sex reversal process, providing a reference for our subsequent trajectory analysis.

### Differential analysis of GSCs during sex reversal in orange-spotted grouper

#### Tracing of GSCs during sex reversal in orange-spotted grouper

**Fig. 2:**
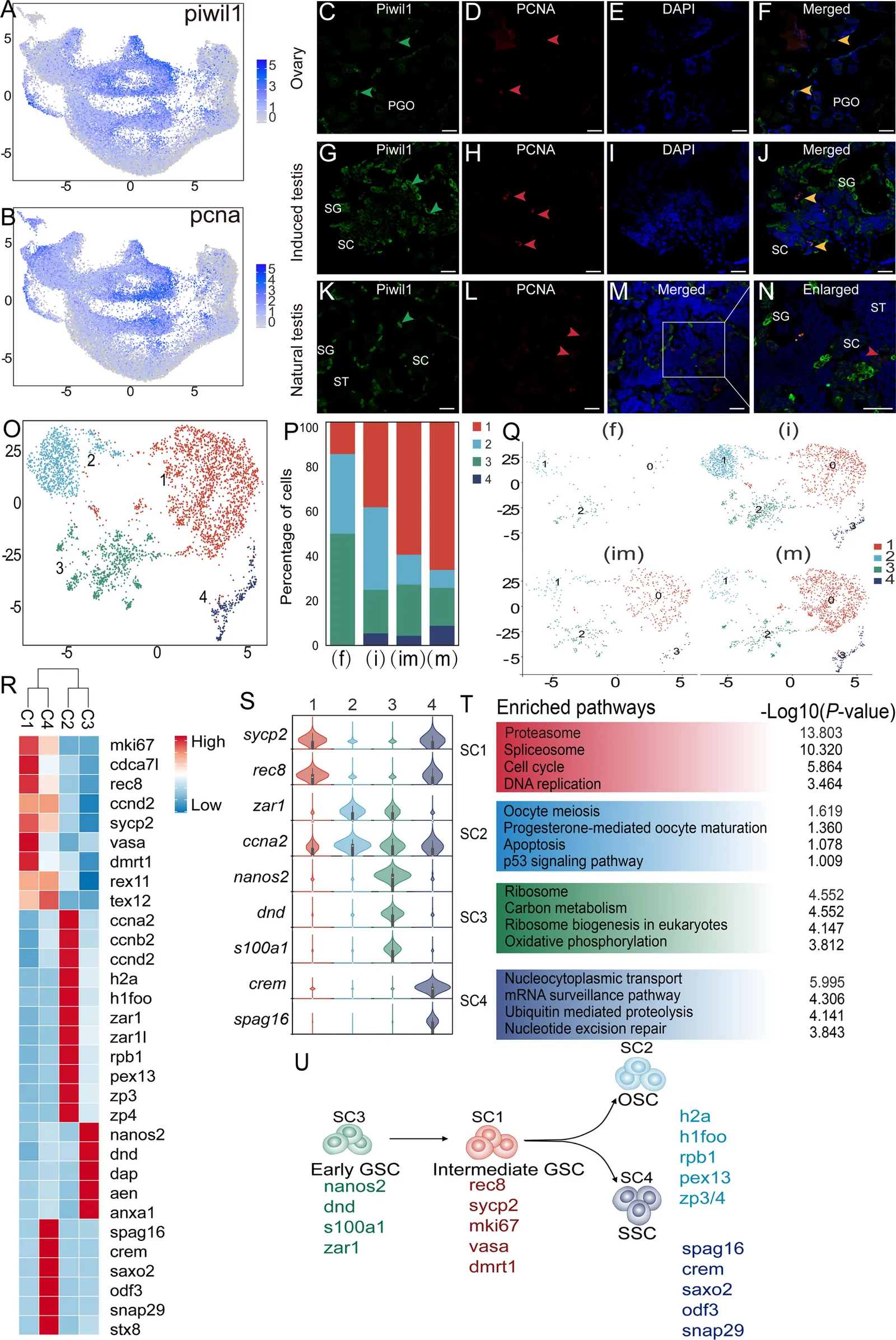
Localization and sub-cluster analysis of GSC (cluster6). UMAP plots of *piwi1l* (**A**) and *pcna* (**B**) in all germ cell clusters. The bluer dots represent the higher expression. **C**–**N** Co-localization of GSC by Piwil1 and PCNA antibodies. **C**–**F** The localization result from ovary without any treatment. **G**–**J** The localization result from MT induced testis. **K**–**N** The localization result from natural testis. **N** The enlarged view of white box in **M**. The green signals were Piwil1 protein, the red signals were PCNA protein, and the blue signals were stained by DAPI. The red arrows represent PCNA signals, the green arrows represent Piwi1l signals, the yellow arrows represent the merged signals. PGO, primary growth stage oocyte; SG, spermatogonia; SC, spermatocyte; ST, spermatotid. Scale bar = 50 μm. **O** UMAP plot of sub-clusters from GSC, with cells colour-coded by computationally determined cell subtypes. **P** Histogram of the percentage of cells in four developmental stages (f, i, im, and m). **Q** UMAP plot showing the distribution of cells in four developmental stages (f, i, im, and m), respectively. **R** Heatmap of selected genes in four subclusters. The redder blocks represent the higher expression. **S** Violin plots of marker genes in four subclusters. **T** KEGG enriched analysis of four subclusters. **U** Schematic diagram of four subclusters during sex reversal. OSC, oogonial stem cell; SSC, spermatogonial stem cell

GSCs possess the characteristics of both germ cells and stem cells, indicating that GSCs express both germ cell marker genes and proliferative cell marker genes. Based on these features and the data from our experiments, we selected the well-known germ cell marker gene *piwil1* and the proliferation marker proliferative cell nuclear antigen gene (*pcna*). Based on the scRNA-seq data, both *piwil1* and *pcna* were highly expressed in GSCs and gonia (Fig. 2A–B). We subsequently assessed the colocalization of Piwil1 and PCNA in the gonads of sex-reversed grouper. The results revealed that the number of GSCs during sex reversal was significantly greater than those in the ovaries and natural testes (Fig. 2C–N). GSCs were primarily located around OG and SG and often appeared as single cells, with very few clustered together. Morphologically, the GSCs were mostly spherical, with a few being spindle shaped. The GSCs were smaller than SG and pSCs and similar in size to the secondary SCs, with a diameter of approximately 10 µm.

#### Heterogeneity and trajectory analysis of GSCs during sex reversal in orange-spotted grouper

In adult fish, GSCs are the origin of all germ cells. After subcluster analysis of the GSC cluster, we found that GSCs were further divided into four distinct subclusters (SC1, SC2, SC3, and SC4) (Fig. 2O). These four subclusters were present in different proportions and were distributed in gonads across four developmental stages (f, i, im, and m) (Fig. 2P–Q). As the process of sex reversal progressed, the number of SC1 significantly increased, whereas the numbers of SC2 and SC3 significantly decreased (Additional file 1: Fig. S4A). SC4 was absent in the ovaries, and its proportion did not significantly differ across the (i, im, and m) stages of the gonads (Additional file 1: Fig. S4A). Further analysis of the DEGs in these four subclusters revealed that SC1 highly expressed proliferation marker genes such as *mki67*; meiosis-related genes such as *cdca7*, *rec8*, *ccnd2*, and *sycp2*; and reproduction-related genes such as *vasa* and *dmrt1*. SC2 highly expressed cell cycle-related genes such as *ccna2*, *ccnb2*, and *ccnd2* and maternal factors such as *h2a*, *h1foo*, *zar1*, *rpb1*, *pex13*, *zp3*, and *zp4*. SC3 highly expressed pluripotency-related genes such as *nanos2* and *dnd*, whereas SC4 highly expressed genes related to spermatogenesis, including *spag16*, *crem*, *saxo2*, *odf3*, and *snap29* (Fig. 2R–S). The transcript counts in SC1 and SC3 were significantly greater than those in SC2 and SC4 (Additional file 1: Fig. S4B), and the proliferation-related genes such as *pcna*, *mki67*, and *mcm4* were highly expressed (Additional file 1: Fig. S4C–E). Moreover, the expression patterns of the DEGs revealed that SC1 and SC4 clustered together, whereas SC2 and SC3 clustered together (Fig. 2R). KEGG enrichment analysis of the DEGs in each subcluster revealed that SC1 was enriched mainly in pathways such as proteasome, spliceosome, cell cycle, and DNA replication; SC2 was enriched in oocyte meiosis, progesterone-mediated oocyte maturation, apoptosis, and the p53 signalling pathway; SC3 was enriched in ribosome, carbon metabolism, ribosome biogenesis in eukaryotes, and oxidative phosphorylation pathways; and SC4 was enriched in nucleocytoplasmic transport, mRNA surveillance, ubiquitin-mediated proteolysis, and nucleotide excision repair pathways (Fig. 2T). These results suggest that SC3 has greater differentiation potential and represents the starting point of GSC differentiation; SC1 has stronger proliferative capacity and represents an intermediate stage of GSC differentiation; and SC2 and SC4 show distinct sexual characteristics and represent the endpoints of GSC differentiation as the starting points of oogonia and spermatogonia, respectively. Therefore, SC3 was classified as early GSCs, SC1 was classified as intermediate GSCs, and SC2 and SC4 were classified as OSCs and SSCs. Based on the UMAP plot, we mapped the differentiation trajectory of GSCs during sex reversal (Fig. 2U).

**Fig. 3:**
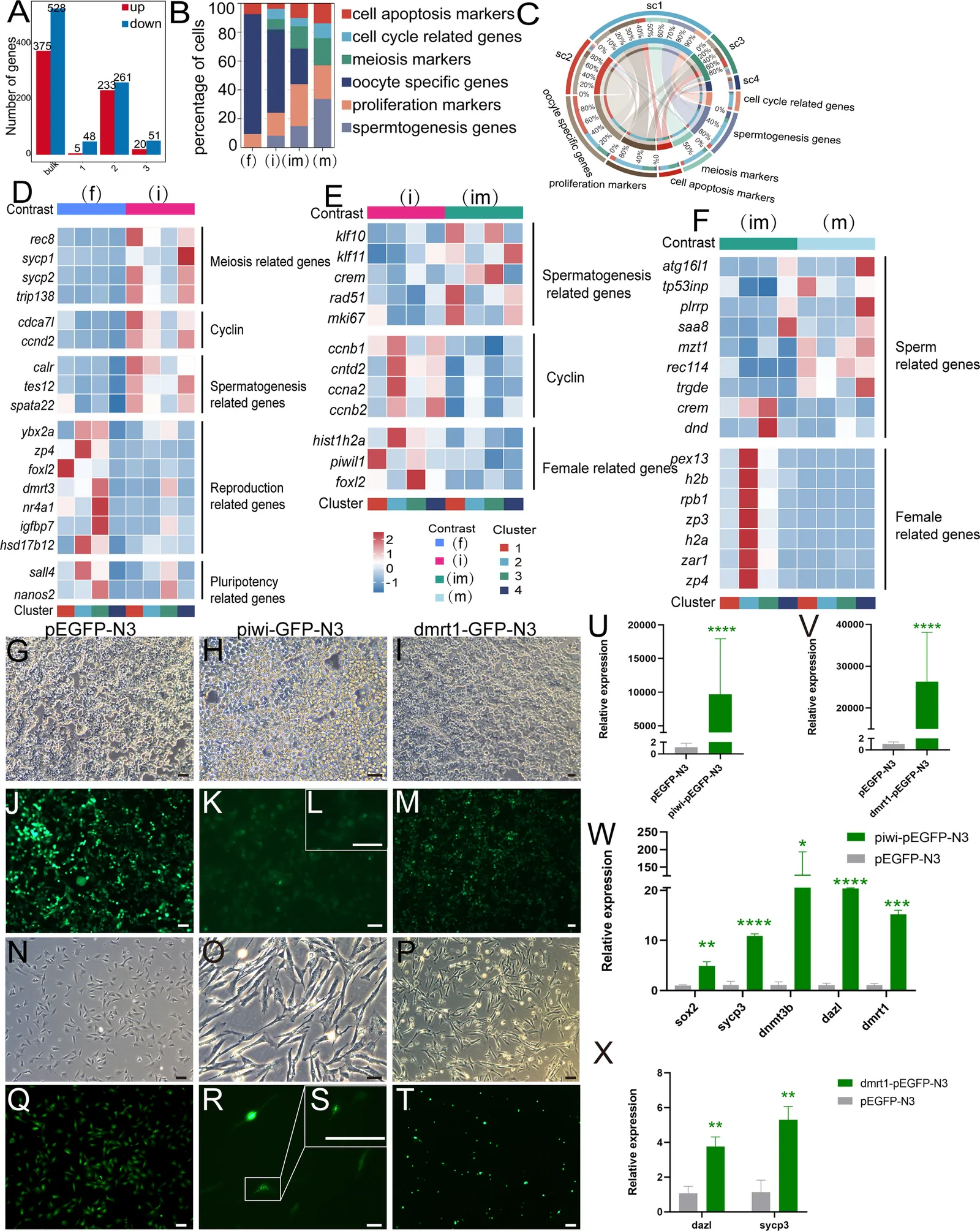
Differential analysis of GSC (cluster6) during sex reversal. **A** Histogram of up (red) and down (blue) differential expressed genes (DEGs) in four subclusters. **B** Histogram of the percentage of six selected gene sets in four developmental stages. **C** Cir-cos plot of six selected gene sets in four GSC subclusters. **D** Comparative analysis of selected genes between four subclusters and (f) group versus (i) group. **E** Comparative analysis of selected genes between four subclusters and (i) group versus (im) group. **F** Comparative analysis of selected genes between four subclusters and (im) group versus (m) group. **G**–**M** The bright and fluorescence field view of transfection with the empty vector (pEGFP-N3, **G**, **J**), *piwil1* (**H**, **K**, and **L**) and *dmrt1* (**I**, **M**) vectors in 293T cells. **N–T** The bright and fluorescence field view of transfection with the empty vector (pEGFP-N3, **N**, **Q**), *piwil1* (**O**, **R**, and **S**) and *dmrt1* (**P**,** T**) vectors in SSC cells of orange-spotted grouper. **L**, **S** The enlarged view of the white box in **K** and **R**. **U**, **V** The relative expression of *piwil1* and *dmrt1* mRNA in overexpression group compared with empty vector group. **W** The relative expression of many genes after over-expression of *piwil1 in* SSC cells. **X** The relative expression of many genes after over-expression of *dmrt1 in* SSC cells. ****, *P *< 0.0001; ***, *P* < 0.001; **, *P* < 0.01; *, *P* < 0.05. SC1, subcluster1; SC2, subcluster2; SC3, subcluster3; SC4, subcluster4; GSC, germ stem cell; SSC, spermtogonial stem cell. Scale bar = 50 μm

To further compare the differences among the different GSC subgroups, we analysed the differential expression changes among the four subgroups in gonads from different developmental stages. The entire GSC cluster included 375 upregulated and 528 downregulated DEGs across the gonads from the four different developmental stages. Specifically, SC1, SC2, and SC3 exhibited 5 upregulated and 46 downregulated, 233 upregulated and 261 downregulated, and 20 upregulated and 51 downregulated DEGs, respectively (Fig. 3A). Six gene sets, including apoptosis markers, cell cycle-related genes, meiosis markers, oocyte-specific genes, proliferation markers, and spermatogenesis genes, were subsequently selected for comparison. First, the percentages of cells expressing the six gene sets differed among the four groups (Fig. 3B). Among the four groups of samples, the proportion of cells expressing oocyte-specific genes and spermatogenesis genes exhibited opposite trends. In addition, the proportion of cells expressing the remaining four gene sets increased during the process of sex reversal. Furthermore, we found that the proportion of cells expressing oocyte-specific genes was greatest in SC2, whereas the proportion of cells expressing spermatogenesis genes was greatest in SC4, indicating that SC2 and SC4 are GSCs with distinct female and male characteristics, respectively. Moreover, the proportions of cells expressing proliferation markers and apoptosis markers were relatively high in SC3 and SC1, respectively, suggesting that SC1 has greater proliferative activity and that SC3 has greater apoptotic activity (Fig. 3C). These findings indirectly validate the accuracy of our definition and differentiation order of the GSC subpopulations.

Comparative analysis of GSCs from untreated ovaries and mid-stage sex-reversed gonads revealed that GSCs in mid-stage sex-reversed gonads significantly overexpressed many genes related to meiosis (*rec8*, *sycp1*, *sycp3*, and *trip128*), the cell cycle (*cdca71* and *ccnd2*), and spermatogenesis (*calr*, *tes12*, and *spata22*). Among these genes, SC1 and SC4 presented the highest expression levels, followed by SC2 and SC3. In contrast, GSCs from untreated ovaries significantly overexpressed reproductive-related genes (*zp4*, *foxl2*, *dmrt3*, *nr4a1*, and *hsd17b12*) and pluripotency-related genes (*sall4* and *nanos2*) (Fig. 3D). These results indicate that during mid-stage sex reversal, the pluripotency of GSCs decreases, leading to greater involvement in spermatogenesis-related activities. As the process of sex reversal progresses, the expression levels of female reproductive-related genes and cell cycle protein genes further decrease, whereas the expression levels of genes related to spermatogenesis and maturation increase. These changes suggest that female reproductive characteristics are lost in late-stage GSCs, particularly when SC1, SC2, and SC3 become more involved in spermatogenic activities (Fig. 3E). Comparing GSCs from artificially induced male fish and natural male fish, we found that the GSCs in natural male gonads overall expressed higher levels of spermatogenesis-related genes, mainly SC1 and SC4, whereas the induced male GSCs, especially SC2, expressed higher levels of female maternal factors and the pluripotency marker gene *dnd* (Fig. 3F). These findings indicate that the GSCs in artificially induced males exhibit higher female characteristics and a lower degree of differentiation.

Given the crucial role of SC1 in the differentiation process of GSCs, we detected changes in the expression of numerous genes, including *dmrt1* and *piwil1*, during the sex reversal process. To determine the roles of both genes in the GSC differentiation process of the orange-spotted grouper, *piwil1* and *dmrt1* were expressed in human embryonic kidney 293T (HEK-293T) cells and our established SSC cell line [51], after which their subcellular localization and changes in the expression of related genes were detected. First, after optimizing the transfection conditions, we overexpressed the *piwil1* and *dmrt1* genes of orange-spotted grouper in the HEK-293T and SSC cell lines (Fig. 3G–T). The localization of these two genes was completely consistent with that in the two types of cells. In particular, the overexpression of the *piwil1* gene yielded the distinct and typical characteristics of liquid‒liquid phase separation in HEK-293T cells and SSCs [52, 53] (Fig. 3K–L, R–S). The subcellular location of *dmrt1* was observed in the nuclei of the two cell lines (Fig. 3M, T). After transfection of the *piwil1* and *dmrt1* vectors, the expression levels of both *piwil1* and *dmrt1* increased by more than 10,000-fold (Fig. 3U, V). We subsequently detected the expression levels of multiple genes. Significant increases in the expression levels of *sox2*, a pluripotency-related gene, and several male germ cell differentiation genes, including *dazl*, *dmrt1*, and *sycp3*, were observed after the overexpression of *piwil1.* Additionally, the expression of the DNA methyltransferase *dnmt3b* was significantly upregulated (Fig. 3W). These results strongly suggest that *piwil1* is closely related to the male fate determination of germ stem cells and epigenetics. After overexpressing *dmrt1*, the expression levels of *dazl*, a germ cell differentiation-related gene, and *sycp3*, a meiosis-related gene, significantly increased (Fig. 3X). These findings indicate that *dmrt1* is associated with the differentiation of GSCs towards the male fate in orange-spotted grouper.

### Integrated comparison of female germ cells during sex reversal

**Fig. 4:**
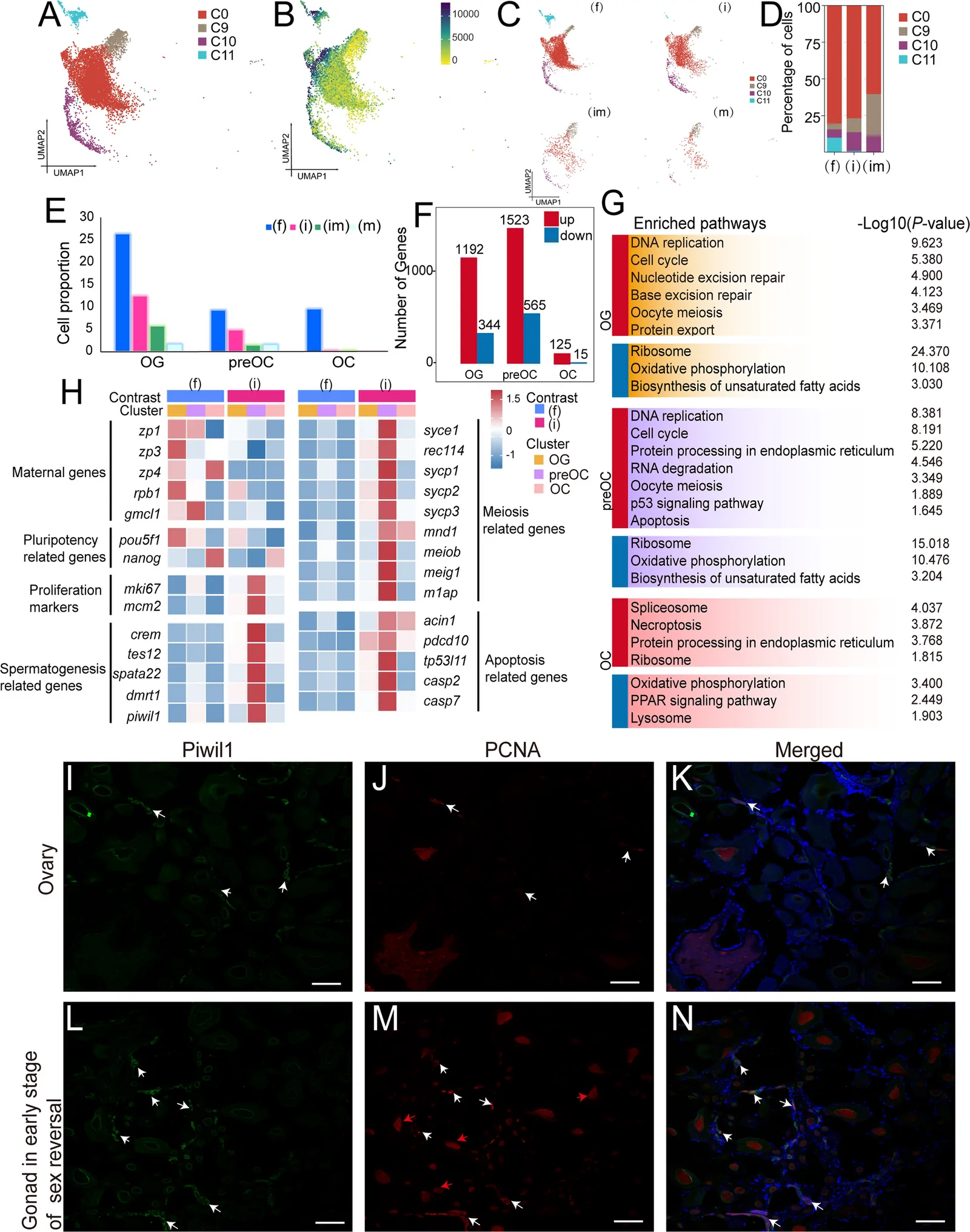
Differential analysis of female germ cells during sex reversal. **A** UMAP plot of four female germ cells clusters (cluster0, 9, 10, 11), with cells colour-coded by computationally determined cell sub-types. **B** UMAP plots of the transcripts level of four female germ cells clusters. **C** UMAP plot showing the distribution of female germ cells clusters in four developmental stages (f, i, im, and m), respectively. **D** Histogram of the percentage of female cell clusters in four developmental stages (f, i, im, and m). **E** Histogram of the percentage of female cell types in four developmental stages (f, i, im, and m). **F** Histogram of up (red) and down (blue) DEGs in three female germ cell types. **G** KEGG enriched analysis of three female germ cell types. **H** Comparative analysis of selected genes between three cell types and (f) group versus (i) group. **I**–**N** Co-localization by Piwil1 and PCNA antibodies in ovary (**I**–**K**) and gonad in early stage of sex reversal (**L**–**N**). The green signals were Piwil1 protein, the red signals were PCNA protein, and the blue signals were stained by DAPI. The red arrows represent PCNA signals, the white arrows represent germ stem cells. Scale bar = 50 μm

To investigate the changes in female germ cells during sex reversal, we conducted a differential comparative analysis of the identified types of female germ cells. First, the female germ cell clusters (Clusters 0, 9, 10, and 11) were reclustered (Fig. 4A). The transcript level of oogonia (Cluster 0) was more abundant (Fig. 4B). The distribution of the four clusters revealed that oocytes were absent in induced testes and natural testes; however, preOCs and OG (Clusters 0, 9, and 10) were present during sex reversal and in natural testes (Fig. 4C–D). These results implied that the testis of hermaphroditic orange-spotted grouper also had the ability to develop oocytes. We subsequently compared the changes in the proportions of female germ cells across the four stages. The results indicated significant decreases in three types of female germ cells (OG, preOCs, OCs) (Fig. 4E). These three cell types underwent dramatic changes during the early stages of sex reversal, with 1192 upregulated DEGs and 344 downregulated DEGs in OG, 1523 upregulated DEGs and 565 downregulated DEGs in preOCs, and 125 upregulated DEGs and 15 downregulated DEGs in OCs (Fig. 4F). The enrichment analysis of these genes revealed that the upregulated DEGs in OG were enriched mainly in pathways related to DNA replication, the cell cycle, nucleotide excision repair, base excision repair, oocyte meiosis, and protein export, whereas the downregulated DEGs were enriched primarily in pathways related to ribosome function, oxidative phosphorylation, and the biosynthesis of unsaturated fatty acids. With respect to preOCs, the upregulated DEGs were enriched in DNA replication, the cell cycle, protein processing in the endoplasmic reticulum, RNA degradation, oocyte meiosis, the p53 signalling pathway, and apoptosis, whereas the downregulated DEGs were enriched mainly in ribosome function, oxidative phosphorylation, and the biosynthesis of unsaturated fatty acids. The upregulated DEGs in OCs were enriched in spliceosome function, necroptosis, protein processing in the endoplasmic reticulum, and ribosome pathways, whereas the downregulated DEGs were enriched in oxidative phosphorylation, PPAR signalling pathway, and lysosome pathways (Fig. 4G). Further analysis of the DEGs revealed that female germ cells in untreated ovaries expressed high levels of maternal genes (*zp1*, *zp3*, *zp4*, *rpb1*, *gmcl1*) and pluripotency-related genes (*pou5f1*, *nanog*). In contrast, female germ cells in early sex-reversed gonads expressed high levels of proliferation marker genes (*mki67* and *mcm2*), apoptosis-related genes (*acin1*, *pdcd10*, *tp53l11*, *casp2*, and *casp7*), meiosis-related genes (*syce1*, *sycp1-3*, *rec114*, *mnd1*, *meiob*, *meig1*, and *m1ap*), and spermatogenesis-related genes (*crem*, *tes12*, *spata22*, *dmrt1*, and *piwil1*) (Fig. 4H). These results indicate that the female germ cells in early sex-reversed gonads clearly differ from those in untreated ovaries, showing reduced female reproductive characteristics and pluripotency, stronger proliferation and apoptosis activities, and greater involvement in spermatogenesis and meiosis. Oocyte apoptosis activity was detected by terminal deoxynucleotidyl transferase dUTP nick end labelling (TUNEL) assay during sex reversal [54]; therefore, we detected proliferation activity using PCNA antibody in the early stage of sex reversal. The colocalization of PCNA and Piwil1 revealed several PCNA signals in normal ovaries (Fig. 4I–K), while many PCNA signals were observed in oocytes and GSCs from the early stage of sex reversal (Fig. 4L–N). Immunofluorescence (IF) results revealed that the proliferation and apoptosis activities of the oocytes increased, especially during the early stage of sex reversal.

### Integrated comparison of male germ cells during sex reversal

**Fig. 5:**
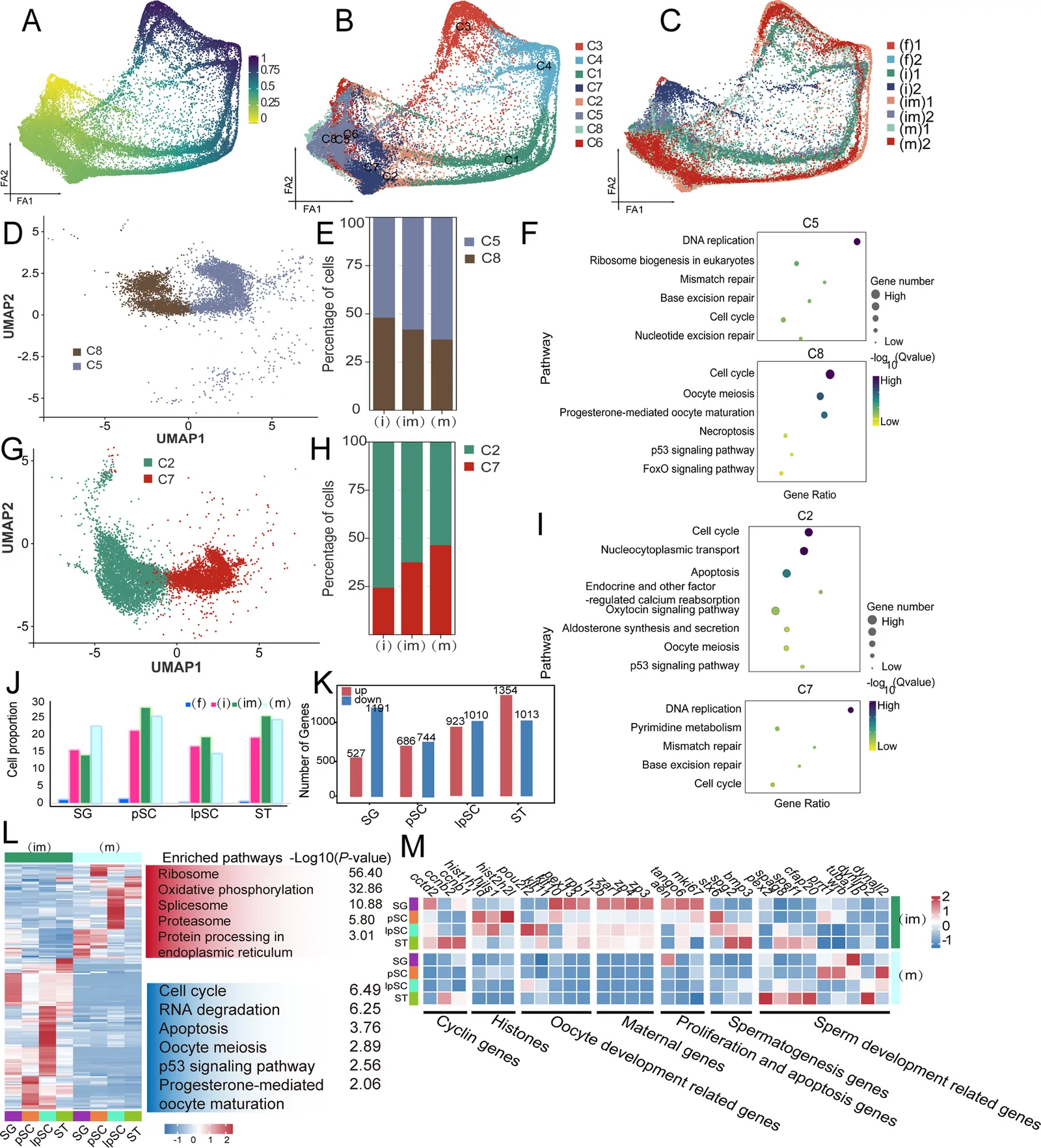
Differential analysis of male germ cells during sex reversal. **A**–**C** PAGA analysis consistently predicts developmental trajectories across eight male germ cell clusters during sex reversal. **A** PAGA-initialized single-cell embeddings in eight male cell clusters. **B** PAGA-initialized single-cell embeddings in eight male cell clusters. **C** PAGA-initialized single-cell embeddings in eight samples. **D** UMAP plot showing the distribution of two types of SG clusters (C5 and C8). **E** Histogram of the percentage of two types of SG clusters in three developmental stages (i, im, and m). **F** KEGG enriched analysis of two types of pSC clusters (C2 and C7). **G** UMAP plot showing the distribution of two types of pSC clusters. **H** Histogram of the percentage of two types of SC clusters in three developmental stages (i, im, and m). **I** KEGG enriched analysis of two types of SC clusters. **J** Histogram of cell proportion of four male germ cells (SG, pSC, lpSC, and ST) in four groups. **K** Histogram of up (red) and down (blue) DEGs in four male germ cell types. **L** KEGG enriched analysis of four male germ cell types between (im) group and (m) group. **M** Comparative analysis of selected genes between four cell types and (im) group versus (m) group. SG, spermatogonia; pSC, primary spermatocyte; lpSC, late primary spermatocyte; ST, spermatid

First, to clearly reflect the differentiation trajectories of all the male germ cells during sex reversal, we performed a pseudotime analysis by partition-based graph abstraction (PAGA) to analyse the potential connections between the cell populations. In the analysis of male germ cells, we observed a single prominent pseudotime trajectory (Fig. 5A), with male germ cells from three different developmental stages distributed sequentially towards SG, pSCs, lpSCs, and STs (Fig. 5B). GSCs serve as the unique starting point for the differentiation of all male germ cells (Fig. 5C). These results indicated that only a portion of GSCs differentiated into female germ cells during the sex reversal process, whereas another portion likely differentiated into male germ cells, which was highly consistent with our earlier results. To distinguish between SG and pSCs, we reanalysed the cell clusters (Clusters 5, 8, 2, and 7). UMAP plots and the proportions of the four clusters were reanalysed across the three developmental stages (f, im, m) (Fig. 5D, E, G, H). The results revealed that the proportion of Cluster 5 increased, whereas that of Cluster 8 decreased during sex reversal (Fig. 5E). Moreover, KEGG enrichment analysis of the four clusters was performed. The DEGs in Cluster 5 were enriched in the DNA replication pathway, and those in Cluster 8 were enriched in the cell cycle and oocyte meiosis pathways (Fig. 5F). During sex reversal, the proportion of Cluster 2 decreased, whereas that of Cluster 7 increased (Fig. 5H). The DEGs in Cluster 2 were enriched in the cell circle, nucleocytoplasmic transport, and apoptosis pathways, and those in Cluster 7 were enriched in the DNA replication pathway (Fig. 5I). These results illustrated that Cluster 5 appeared earlier than Cluster 8 did, and Cluster 7 appeared earlier than Cluster 2 did.

To investigate the changes in male germ cells during sex reversal, we conducted a differential comparative analysis of the defined types of male germ cells. There were no significant differences in the proportions of SG, pSCs, SCs, or STs across the four gonadal stages (Fig. 5J). These three cell types underwent dramatic changes during sex reversal, with SG showing 527 upregulated DEGs and 1191 downregulated DEGs, pSCs showing 666 upregulated DEGs and 744 downregulated DEGs, lpSCs showing 923 upregulated DEGs and 1010 downregulated DEGs, and STs showing 1354 upregulated DEGs and 1013 downregulated DEGs (Fig. 5K). The enrichment analysis of the DEGs revealed that pathways related to ribosome function, oxidative phosphorylation, the spliceosome, the proteasome, and protein processing in the endoplasmic reticulum were significantly enriched in natural male fish, whereas pathways related to the cell cycle, RNA degradation, apoptosis, oocyte meiosis, p53 signalling, and progesterone-mediated oocyte maturation were significantly enriched in induced male fish (Fig. 5L).

Further analysis of the DEGs revealed that male germ cells in induced male fish presented significantly increased expression levels of cell cycle-related genes (*cctd2*, *ccnb1*, and *ccnb2*), histone genes (*hist1h1d*, *hils1*, and *hist2h2l*), oocyte development-related genes (*pou2f2*, *klf11*, *klf10*, *pex13*, and *rpb1*), maternal genes (*h2b*, *zar1*, *zp3*, and *zp4*), and proliferation- and apoptosis-related genes (*aen*, *tango6*, and *mki67*), as well as spermatogenesis-related genes (*stx6*, *spg2*, and *bmp3*). In contrast, the expression levels of spermatogenesis-related genes (*pex2*, *spag8*, *spef1*, *cfap20*, *prrt1*, *wrb*, *tuba1b*, *dynlrb2*, and *dynall2*) were significantly increased in male germ cells in natural male fish (Fig. 5M). These series of results indicate a clear difference between the male germ cells of natural and induced males, with the SG of induced males exhibiting more characteristics of female germ cells, displaying more active proliferation and apoptosis, and becoming more involved in spermatogenic activities. Conversely, the male germ cells of natural males lost their female germ cell characteristics, showing lower levels of proliferation and apoptosis, and were more involved in spermatogenesis activities.

### Heterogeneity and trajectory analysis of gonadal somatic cell during sex reversal in orange-spotted grouper

**Fig. 6:**
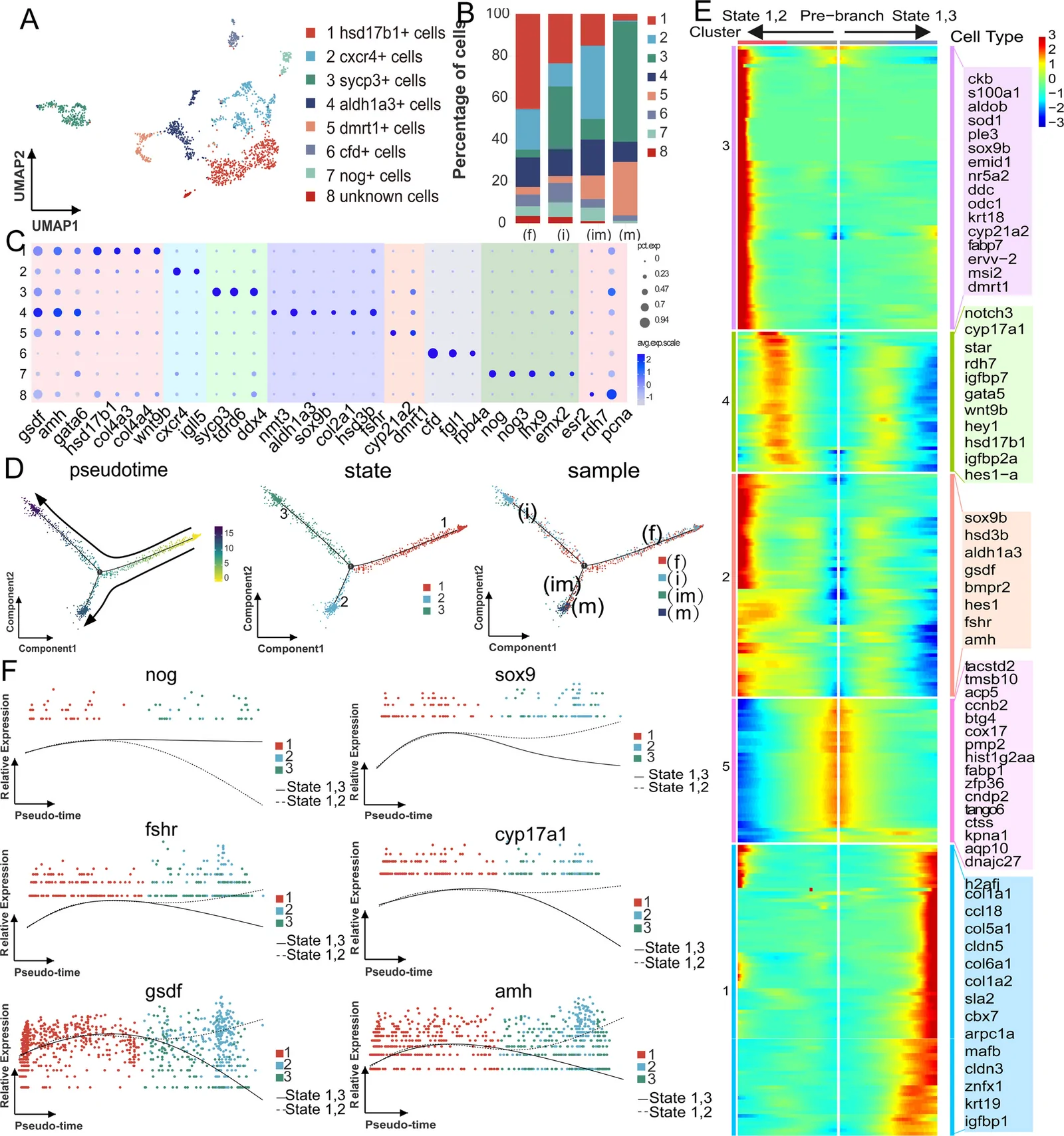
Subcluster and pseudotime trajectory analysis of gonadal somatic cells (cluster9) during sex reversal. **A** UMAP plot of sub-clusters from cluster9, with cells colour-coded by computationally determined cell subtypes. **B** Histogram showing the distribution of cells in four gonadal stages (f, i, im, and m). **C** Dot-plot showing the relative expression of marker genes in eight sub-clusters. The bluer dots represent the higher expression. **D** Pseudotime trajectory plots of gonadal somatic cells in different subclusters and samples. **E** Heatmap of significantly changed genes. The heatmap shows the expression patterns of genes that significantly changed along the pseudotime trajectory. **F** Spline plots of representative gene expression dynamics in gonadal somatic cells populations

Cluster 9 was defined by the high expression levels of three marker genes, namely, *gsdf*, *amh*, and *gata6* (Fig. 1E). These genes have been found in various fish species, with studies indicating that *gsdf* is expressed in granulosa cells in ovaries and Sertoli cells in testes [4, 55]; *amh* is expressed in cortical vesicle-stage oocytes and Sertoli cells in orange-spotted grouper testes [24]; and *gata6* is expressed primarily in follicular cells in ovaries and Leydig cells in testes [56]. Therefore, the high expression levels of these three genes in Cluster 9 suggested that the group was not composed of a single cell type but included at least granulosa cells, Sertoli cells, and Leydig cells, which were defined as gonadal somatic cells. When we performed subcluster analysis on gonadal somatic cells, we identified a total of eight subclusters (Fig. 6A). The distribution of these subclusters (scs) varied across different stages of gonadal development (Fig. 6B). The abundances of sc1 and sc2 were greater in the ovaries, and their proportions decreased as the sex reversal process progresses, whereas the abundances of sc5 and sc7 were significantly greater in the testes than in the other gonads. Furthermore, marker genes of eight subclusters were analysed (Fig. 6C). First, sc6, characterized by high expression levels of *nog* (noggin), *nog3* (noggin3), *lhx9*, and *emx2*, was defined as the precursor of granulosa cells (Fig. 6C, Additional file 1: Fig. S5A–D). These genes have been previously identified as markers for follicular cell precursors and in zebrafish [57]. Similarly, these genes were also defined as markers for follicular cell precursors in gonadal data from the sex differentiation process of orange-spotted grouper [43]. Sc1 and sc4 universally exhibited high expression levels of *gsdf*, *amh*, and *gata6*. Moreover, some cells in sc4 and sc5 highly expressed *star* and *hsd3b1*, which are specific markers for Leydig cells, especially in sc5 (Additional file 1: Fig. S5E, F). Sc4 also highly expressed *fshr* and *sox9a*, which are markers of Sertoli cells (Additional file 1: Fig. S5G, H) [58–60]. Additionally, some cells in sc2 expressed *cyp19a1a*, a marker of granulosa cells [23] (Additional file 1: Fig. S5I).

After the interference from sc3 and sc8 was excluded, the remaining six subclusters were subjected to pseudotime trajectory analysis. Analysis of the pseudotime trajectory revealed three major branching points, thereby segregating the cells into three discrete states. Notably, with increasing processing time, cells transitioned from earlier states (state 1) to more advanced states (state 3) along the pseudotime trajectory. The four samples were distributed across three distinct branches. The samples in group (f) were located at the origin of the pseudotime trajectory, whereas the samples in group (i) were distributed along two distinct branches that diverge in different directions from groups (im) and (m) (Fig. 6D). To delineate DEGs along the pseudotime trajectory, we conducted clustering analysis on the expression profiles of all cells encompassed within this trajectory. The identified DEGs were subsequently stratified into five distinct clusters in accordance with their temporal expression trends (Fig. 6E). The significant expression levels of many DEGs were related to the synthesis and secretion of steroid hormones (e.g., *hsd3b*, *cyp17a1*, *sox9*, *fshr*, *gsdf*, and *amh*), highlighting their potential roles in the differentiation of gonadal somatic cells during sex reversal. The expression levels of these genes were upregulated in states 1 and 2, as was *nog* expression (Fig. 6F). These findings indicated that the gonadal somatic cells in both the testes and ovaries consist of various types, sharing common features but also exhibiting their own unique characteristics. This pseudotime analysis also provided insights into the origins of Sertoli and Leydig cells in the testes.

**Fig. 7:**
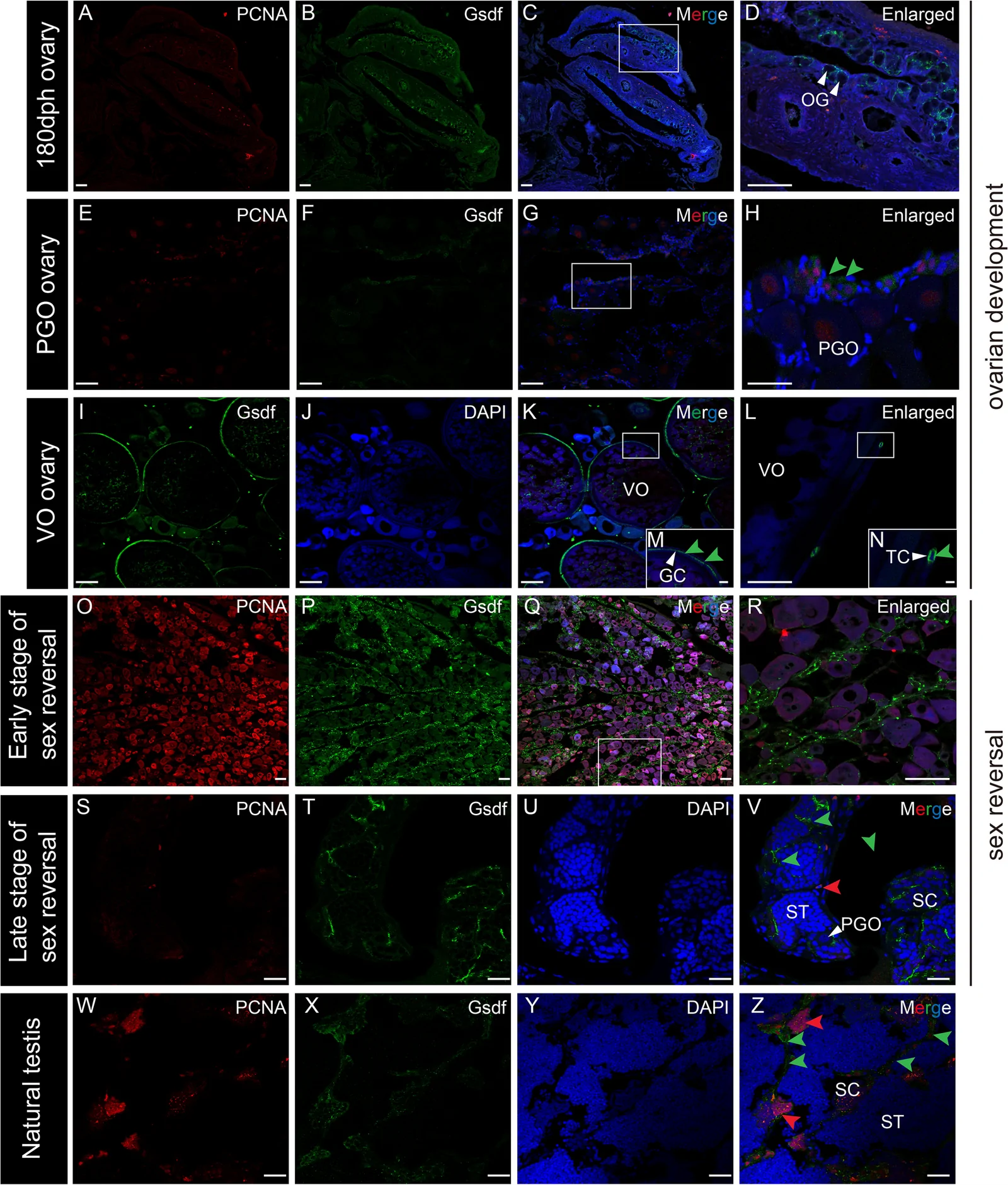
Localization of Gsdf antibodies in differential developmental gonads. **A**–**D** The localization result from 180 dph ovary after sex differentiation with many oogonia. **E**–**H** The localization result from ovary with many PGOs. **I**–**L** The localization result from ovary with plenty of VOs. The red signals were PCNA protein, the green signals were Gsdf protein, and the blue signals were stained by DAPI. **D**, **H**, **M**, **N** The enlarged views of white boxes in **C**, **G**, **K**, and **L**. **O**–**R** The localization result from gonad in early stage of sex reversal with a majority of oocytes and a small number of male germ cells. **R** The enlarged view of the white box in Q. **S-V** The localization result from gonad in late stage of sex reversal, and the gonad was filled with plenty of male germ cells. **W**-**Z** The localization result from natural male testis. The red signals were PCNA protein, the green signals were Gsdf protein, and the blue signals were stained by DAPI. The green arrows represent Gsdf signals, the red arrows represent PCNA signals, and the white arrows represent different cells. PGO, primary growth stage oocyte; VO, vitellogenesis oocyte; GC, granulosa cell; TC, theca cell; SC, spermatocyte; ST, spermatid. Scale bar = 50 μm

To verify the accuracy of the marker genes, we prepared a polyclonal antibody against Gsdf in orange-spotted grouper. Using this antibody, we conducted expression localization analysis on gonads at different developmental stages. First, when the granulosa and theca cells had not yet formed, Gsdf was expressed primarily in the interstitial cells surrounding the oogonia at 180 dph (days post hatch) in the ovaries after sex differentiation was complete (Fig. 7A–D). In ovaries filled with primary growth-stage oocytes, the Gsdf signal weakened and was concentrated in a small number of gonadal somatic cells (Fig. 7E–H). As yolk granules accumulated, granulosa and theca cells formed a double-layered membrane structure around the oocytes, and Gsdf was expressed mainly in the granulosa cells surrounding the yolk-laden oocytes (Fig. 7I–K, M), with weak signals detected in the yolk granules and some theca cells (Fig. 7L, N). Upon artificial induction of sex reversal, in the early-stage gonads during sex reversal, we found that Gsdf was expressed not only in the ring of granulosa cells surrounding the oocytes but also in many somatic cells (Fig. 7O–R). In the late-stage gonads during sex reversal, Gsdf was still expressed in the ring of follicular cells around the oocytes and was specifically expressed in Sertoli cells, as also observed in the testes (Fig. 7S–V). In natural testes, Gsdf was expressed in Sertoli cells (Fig. 7I–L). During sex reversal, the PCNA signal was concentrated in GSCs and SG, which did not overlap with the Gsdf-positive cells but rather surrounded them (Fig. 7W–Z).

## Discussion

In this study, we conducted single-cell transcriptomic analysis of the gonads of MT-induced groupers before and after sex reversal. We obtained a high-resolution gonadal cell atlas of hermaphroditic fish during the sex reversal process. These results not only reflected the transcriptional level changes in all the gonadal cells during sex reversal in groupers but also revealed the differentiation trajectories of all the cells as well as specific cells. This study identified the origin of male germ cells during the sex reversal process, providing new insights into the molecular mechanisms of sex reversal in hermaphroditic fish. Additionally, by comparing the differences in gonadal cells between artificially induced male fish and naturally sex-reversed male fish, we obtained new insights into the evaluation of artificially induced male fish.

In this study, gonadal samples during sex reversal were obtained for at least 6 weeks by in vivo implantation. To better simulate natural sex reversal, we selected orange-spotted groupers weighing more than 2 kg for experimentation. Considering experimental costs and operational complexity, we collected samples from 10 fish per group weekly. However, challenges such as asynchronous gonadal reversal and wound infection-related mortality were observed during the MT-induced sex reversal experiments. Therefore, after determining the approximate developmental stages of gonads during sex reversal, we implemented pooled sampling (*n* = 3) to minimize individual variability. Although only two biological replicates were conducted per developmental stage, our scRNA-seq data demonstrated consistent quality, with continuous changes in multicell cluster proportions across all four developmental stages. Moreover, the highly expressed genes in each cell cluster were consistent with published data [43, 50] (Fig. 1). These results confirm the feasibility of our sampling methodology and the reliability of the obtained scRNA-seq data.

Spermatogenesis of artificially induced male and naturally sex-reversed male groupers is conserved similarly to that of mammals. In this study, we defined male germ cells (SG, pSCs, lpSCs, and STs) from artificially induced and naturally sex-reversed testes by typical markers, such as *dmrt1*, *calr*, *rec8*, *sycp1-3*, and *crem*. Other somatic cells, such as Sertoli cells and Leydig cells, were identified based on *gsdf*, *amh*, and *gata6*. These results indicate that signature genes of distinct cell types established in gonochoristic mammals could serve as markers for nonmammalian hermaphroditic vertebrates. Thus, the cell types present in the gonads were similar to those found in mammals [61]. Furthermore, the expression profiles of the gonadal cells from artificially induced male and naturally sex-reversed male groupers were highly consistent with our previously established database of naturally occurring male groupers [50]. Additionally, in an unsupervised pseudotime analysis, SG, SCs, and STs followed a continuous trajectory. These results indicate that the essential features of spermatogenesis are highly conserved across vertebrate taxa. The features of male germ cells were similar between induced testes and natural testes.

After an in-depth comparison of both testis types, we identified several differences between hormone-induced testes and natural testes. First, natural testes presented greater proportions of more mature male germ cells, such as SCs (Cluster 3) and pSCs (Cluster 7; Fig. 5E–F). Additionally, natural testes contained greater numbers of intermediate and male-oriented GSCs (Fig. 2U). These GSCs expressed high levels of genes related to spermatogenesis, meiosis, the cell cycle, and apoptosis (Fig. 3B). In contrast, the DEGs of male germ cells in natural testes were significantly enriched in pathways such as ribosome, oxidative phosphorylation, spliceosome, proteasome, and protein processing in the endoplasmic reticulum (Fig. 5L). These findings collectively indicated that male germ cells are more mature in natural testes and that GSCs possess bidirectional differentiation potential but consistently differentiate towards male-oriented pathways. These results are related to the natural males selected. Because we cannot monitor gonadal development in live fish, we can only determine sex by observing semen expulsion ability. The presence of ejaculate indicates that the male fish are in the reproductive stage and have high levels of sperm maturation. Hormone-induced males develop a spermatogenic microenvironment within a short period, which can quickly revert to a female-like state after hormone withdrawal. This highly plastic state inevitably means that hormone-induced testicular maturation occurs at significantly lower levels than does natural testicular development. However, this deficiency can be compensated by extending the duration of hormone treatment.

As hermaphroditic fish, groupers have long been used as a model for studying sex reversal and sex differentiation. Many genes, such as *dmrt1* [26], *amh* [24], have been identified to play important roles during sex reversal. Our team has focused on the study of grouper, particularly orange-spotted grouper, and has constructed various genomic databases for this species [28, 50, 54]. We have also screened and identified numerous marker genes for orange-spotted grouper germ cells, including *vasa*, *dazl*, *nanog*, *dnd*, *slbp2*, and *zbtb40* [31–33, 54]. These research foundations provide valuable tools for the identification of cell types in this study. To further investigate the changes in all cell types during sex reversal in orange-spotted groupers, we employed the currently popular single-cell transcriptome sequencing technology, combined with our previous experience in preparing cell suspensions. We successfully obtained high-quality scRNA-seq data, which ensured the reliability of our subsequent data analysis. Throughout the exploration of the molecular mechanisms underlying sex reversal in orange-spotted groupers, two major questions have always troubled us: (1) Where do the newly formed male germ cells come from during sex reversal? (2) Where do Sertoli cells and Leydig cells originate from in induced testes?

Through a series of data analyses in this study, we can now confidently answer these questions. (1) First, through UMAP analysis and pseudotime analysis, we were surprised to find a common intersection between female and male germ cells. The cell cluster was defined as GSCs based on their expression characteristics (Fig. 1G). Subgroup analysis revealed that these GSCs can be divided into four distinct subclusters (Fig. 3U). When we analysed the expression characteristics and differentiation trajectories of these four subclusters, we found that these subclusters clearly differentiated towards both sexes, with an intermediate state required for the transition. In the ovary, there are no SSCs and a very low proportion of intermediate-state GSCs compared with those in the other developmental stages (i, im, m). SSCs begin to emerge during sex reversal. However, mature testes contain OSCs, SSCs, and a relatively high proportion of intermediate-state GSCs. These results indicate that all male germ cells originate from male GSCs during sex reversal and that these SSCs share a common precursor with OSCs. These findings also explain why the orange-spotted grouper can only develop into females initially, with only approximately 5% of females reverting to males in the natural state. In contrast, male individuals can mostly transform into females when social and physiological conditions change. These findings provide new insights into the reproductive characteristics of orange-spotted groupers, which undergo female-to-male sex reversal. (2) Numerous studies have reported that Sertoli cells originate from gonadal cord cells in the gonadal ridge during embryonic development [62, 63]. In adult fish, Sertoli cells possess a certain capacity for proliferation [64]. Some researchers believe that a stem cell population of Sertoli cells may exist that can differentiate into mature Sertoli cells to maintain the normal function of the testis [65]. However, where do Sertoli cells come from during sex reversal in hermaphroditic fish? Based on our data analysis, we believe that both granulosa cells and Sertoli cells originate from a common precursor cell. During the juvenile stage, before they form, granulosa cells exist as interstitial cells scattered around the oogonial cells and oocytes (Fig. 7D, H, L). As the oocytes mature, granulosa cells gradually form and surround them. During the sex reversal process in orange-spotted groupers, the number of interstitial cells increases significantly, which may be the source of Sertoli cells in the testes. These cells are also very likely to be the origin of Leydig cells.

**Fig. 8:**
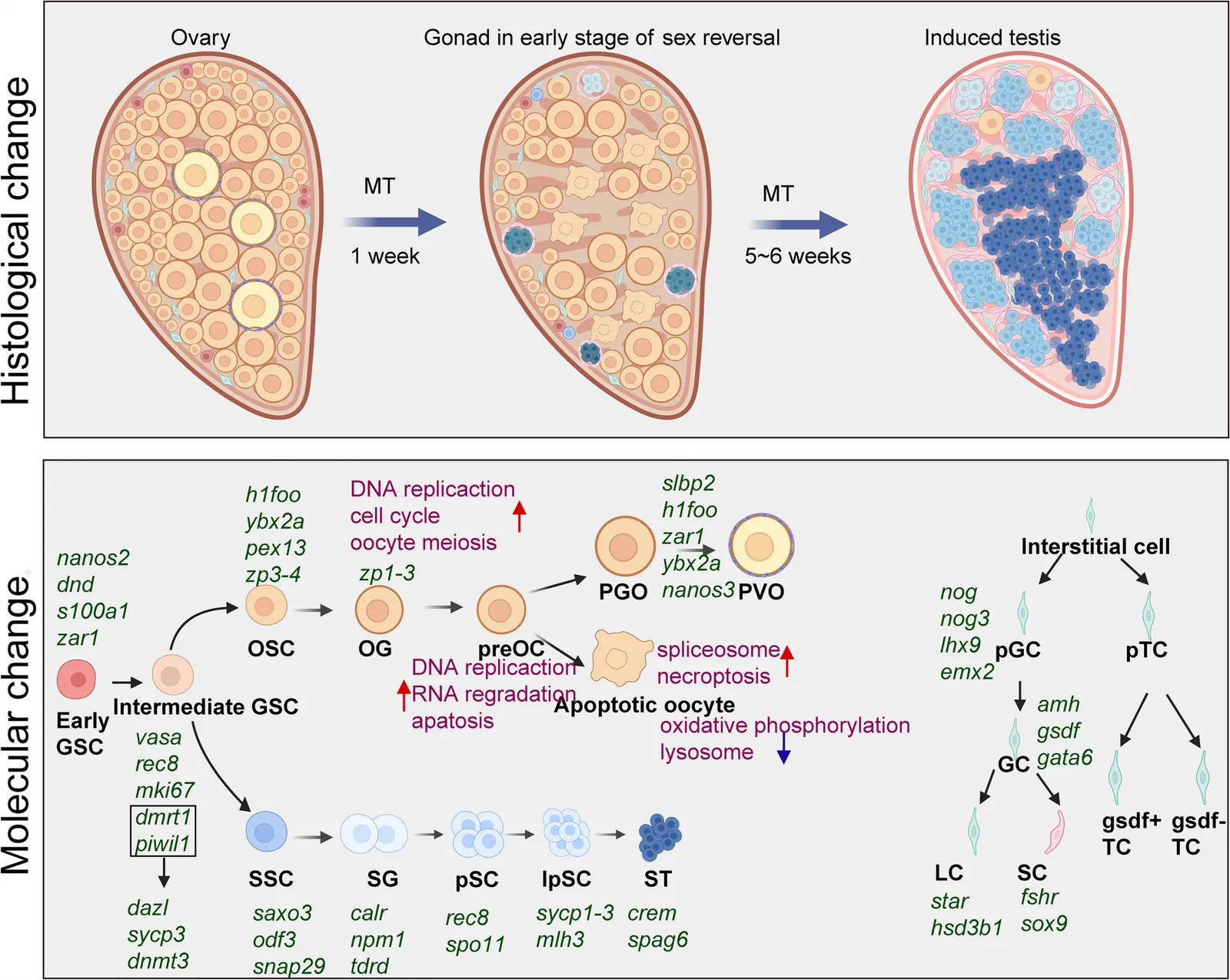
Model of sex reversal induced by hormone in orange-spotted grouper created with BioRender.com. The upper portion of the figure illustrates the histological changes in gonads during sex reversal. Initially, starting from the typical structure of the ovary, gonad progresses through the early phase of sex reversal where oocytes undergo atrophy and apoptosis, and male germ cells emerge. By the late stage of sex reversal, almost all oocytes have disappeared from the gonads, which are now filled with a large number of various types of male germ cells. The lower half of the figure illustrates molecular-level changes in gonadal cells during sex reversal. The differentiation of GSCs into male and female gametes exhibits distinct characteristics at each developmental stage. Dark bold text indicates different cell types, green italicized text represents characteristic genes at specific stages, purple text denotes significantly enriched signaling pathways, with red arrows indicating upregulation and green arrows representing downregulation. MT, 17alpha-methyltestosterone; GSC, germ stem cell; OSC, oogonial stem cell; SSC, spermtogonial stem cell; OG, oogonia; OC, oocyte; PGO, primary growth stage of oocyte; PVO, cortical-alveolus oocyte; SG, spermatogonia; pSC, primary spermatocyte; lpSC, late primary spermatocyte; ST, spermatid

The orange-spotted grouper is a hermaphroditic fish species that undergoes female-to-male sex reversal during its life cycle, with sex reversal being a common phenomenon. To explore the molecular mechanisms underlying sex reversal in orange-spotted groupers, we artificially induced sex reversal and compared the gonadal scRNA-seq data before, during, and after the sex reversal process. Owing to the limitations of the 10 × Genomics sequencing method, cells with a diameter greater than 40 μm (mainly PGOs and PVOs) were filtered out after the preparation of the cell suspension. As a result, the number of female germ cells in the entire sequencing dataset was much lower than that of male germ cells. Additionally, because the ovaries collected were still at a developmental stage dominated by PGOs, the numbers of granulosa cells and theca cells in the gonads, especially theca cells, were still very low. Therefore, there was no clear cluster of theca cells in the final data (Fig. 1F). Nevertheless, the data from this experiment still fulfil the original intent of our experimental design to investigate the molecular mechanisms of sex reversal. Through this comparison, we have outlined the dynamic changes in various cells during the sex reversal process. In the early stage of sex reversal, genes related to meiosis, the cell cycle, and spermatogenesis are highly expressed, causing GSCs to begin transitioning into SSCs. At this stage, the female characteristics and pluripotency of GSCs are gradually lost. Later, in the final stage of sex reversal, the SSC characteristics of GSCs become more pronounced, and the female characteristics continue to diminish. Compared with those in artificially induced males, GSCs in natural males are more masculinized and retain the lowest degree of female germ cell characteristics, which may be related to the greater maturity of natural males. After the formation of SSCs, they rapidly differentiate into large numbers of SG, pSCs, lpSCs, and STs. The gonads are quickly populated by a large population of male germ cells, resulting in the characteristic structure and features of the testes. During the sex reversal process, the state of female germ cells is disordered. Compared with normal females, OG and preOCs gradually lose the characteristics of female germ cells. Moreover, on the one hand, preOCs maintain a high proliferative state, but on the other hand, they are in a state close to apoptosis and atrophy. These results provide a detailed description of the entire process of molecular changes and differentiation trajectories in germ cells and gonadal cells during sexual reversal. Based on these results, we constructed a schematic diagram illustrating gonadal cell changes and molecular expression alterations during sex reversal in orange-spotted grouper (Fig. 8).

## Conclusions

During sex reversal, gonads at four different stages were isolated for scRNA-seq analysis. The scRNA-seq data provided a blueprint of the dynamics and differences of all the gonadal cells during sex reversal. Moreover, we outlined the trajectories of all the gonadal cells and traced them back to the origins of male germ cells, Sertoli cells and Leydig cells. These results provide valuable data for understanding the regulatory mechanism of sex reversal in orange-spotted grouper and other protogynous hermaphroditic species.

## Methods

### Animals

Orange-spotted groupers were raised at the Guangdong Marine Fishery Experiment Center (Huizhou 516081, Guangdong, China). The fish were kept in indoor pools, and the water temperatures were controlled at 22.7–27.8 °C. Fish gonads were sampled from different periods to confirm the developmental stages by histological morphology and prepare single-cell suspensions. All the fish were anaesthetized with MS222 and then sacrificed.

### Artificially induced sex change by MT implantation

In this study, artificially induced sex reversal was carried out by 17 alpha methyl testosterone (MT; Sigma‒Aldrich, USA) treatment. The fabrication of the slow-release strips and the steps of MT implantation were performed according to our previous paper with minor modifications [31]. The fish (body weight, 2.0 ± 0.25 kg; body length, 32.7 ± 10.3 cm) were divided into two groups: a sham or control group (*n* = 60) and an MT implantation group (*n* = 60). The dosage of MT was 5 mg/kg body weight. Before implantation (Week 0), the gonadal tissues of the fish were collected randomly (*n* = 6). After MT implantation, ten fish were sampled randomly every week from the two groups until 9 weeks.

### Haematoxylin‒eosin (H&E) staining

The fresh gonads were fixed in Bouin’s solution for 24 h. Afterwards, the tissues were dehydrated in an orderly manner using gradient alcohol and embedded in paraffin. The tissues were further cut into 5μm-thick slices. Finally, the nucleus and cytoplasm were stained with haematoxylin and eosin, and the sections were observed by light microscopy (Nikon IQ50, Japan) to classify the gonadal stages.

### Real-time quantitative PCR validation

Real-time quantitative PCR (RT‒qPCR) was used to detect changes in the expression of marker genes during sex reversal. Total RNA from 0, 3, 6, 7, and 9 weeks after sex reversal was extracted using TRIzol (Thermo, USA), and RNA quality was subsequently detected with a 2100 Bioanalyzer (Agilent, CA, USA) and quantified using an ND-2000 (Nanodrop Technologies, DE, USA). For real-time PCR, RNA was reverse transcribed to cDNA with a ReverTra Ace-α First-Strand cDNA Synthesis Kit (TOYOBO, Japan). The reverse transcription process was as follows: 37 °C for 15 min, 98 °C for 5 min, and 4 °C for 5 min. The amplification regime was 35 cycles of 94 °C for 20 s, 55 °C for 10 s, and 72 °C for 20 s, followed by further amplification at 72 °C for 5 min. Real-time PCR was performed on an ABI 7900 real-time PCR System using the SYBR Green I Kit (TOYOBO, Japan). All mRNA quantification data were normalized to that of *β-actin* and are presented as a relative control group. The specific primers used in this study are listed in Additional file 2: Table S3.

### Preparation of gonadal single-cell suspensions

The gonads from three fish were combined into a single sample. A mixture of gonads from the control group was dissociated for scRNA-seq, with two repetitions (*n* = 2). As the process of sex reversal progresses, three gonadal tissue samples were mixed at the intermediate stage and late stage of sex reversal and then digested into a single-cell suspension sample for scRNA-seq. The digestion method was described in our previously published article [43]. Briefly, the gonadal samples were cut into pieces and then cleaned twice with 1 × Hank’s balanced salt solution. Next, the samples were centrifuged at 1000 × g for 3 min and incubated with 500 U/mL collagenase IV (diluted with DPBS buffer without calcium or magnesium ions) at 37 °C for 2 h. Then, the samples were centrifuged at 1000 × *g* for 3 min, after which they were filtered through a 40-µm strainer (Corning, USA). The cell numbers of the suspensions were calculated using a blood counting chamber, after which the cells were stained with 0.4% trypan blue to estimate cell viability. The suspensions (cell viability > 85%) were then used for further scRNA-seq.

### Library preparation and scRNA-seq

ScRNA-seq libraries were constructed using Chromium Next GEM Single-Cell 3’ Reagent Kits v3.1 (10 × Genomics, USA). The prepared suspensions were used to generate single-cell Gel Beads-In-Emulsions (GEMs) by loading onto a 10 × Genomics GemCode Single-cell. Upon dissolution of the single-cell 3’ gel bead in a GEM, primers containing (i) an Illumina® R1 sequence (read 1 sequencing primer), (ii) a 16 nt 10 × barcode, (iii) a 10 nt UMI, and (iv) a poly-dT primer sequence were released and mixed with the cell lysate and Master mix. The GEMs were incubated to produce barcoded, full-length cDNA from polyadenylated mRNA. Afterwards, the cDNA was amplified by PCR to generate a sufficient mass for library construction. R1 (read 1 primer sequence) was added to the molecules during GEM incubation. P5, P7, a sample index, and R2 (read 2 primer sequence) were added during library construction via end repair, A-tailing, adaptor ligation, and PCR. The final libraries contain the P5 and P7 primers used in Illumina bridge amplification.

The single-cell 3’ protocol produced Illumina-ready sequencing libraries. A single-cell 3’ library comprised standard Illumina paired-end constructs that began and ended with P5 and P7. The single-cell 3′ 16 bp 10 × barcode and 10 bp UMI were encoded in Read 1, whereas Read 2 was used to sequence the cDNA fragment. Sample index sequences were incorporated as the i7 index read. Read 1 and Read 2 were standard Illumina® sequencing primer sites used in paired-end sequencing.

### Data quality control and gene expression quantification

Raw BCL files were converted into FASTQ files using Cell Ranger software (version 5.0). After mapping to the orange-spotted grouper genome [16], the reads that uniquely intersected at least 50% of an exon were considered for UMI counting. Valid barcodes were identified using the EmptyDrops method [66]. Seurat (version 3.1.1) was used to perform quality control (QC), analysis, and exploration of the scRNA-seq data. Cells with UMIs (≥ 17,000), mitochondrial genes (≥ 10%), ≤ 230 detected genes, or ≥ 2200 detected genes were filtered out. After unwanted cells were removed from the dataset, the global-scaling normalization method “LogNormalize” was used to normalize the gene expression measurements for each cell by the total expression, multiplied by a scale factor (10,000 by default), and log-transformed the result by the formula as follows: gene expression level = log(1 + (UMI A ÷ UMI Total) × 10,000).

To minimize the batch effect, a canonical correlation analysis (CCA) was used [67]. The scaled expression matrix was used for dimensional reduction by principal component analysis (PCA). Afterwards, the significant principal components (PCs) were identified by strong enrichment of low-*p* value genes, and the cells were subsequently clustered by Seurat based on their PCA scores [68]. To cluster the cells, the modularity optimization technique SLM was applied to iteratively group the cells together to optimize the standard modularity function [69]. In this experiment, we chose a resolution of 0.2 as the clustering parameter to ultimately identify 25 clusters. UMAP was subsequently performed to visualize the data in two-dimensional space.

### Differential expression analysis

To identify enriched genes in a specific cluster, the median expression patterns of all clusters were calculated, and the expression value of each gene in the cluster was subsequently compared with that of the remaining cells using the Wilcoxon rank-sum test [70]. The significantly upregulated genes were identified using several criteria, including (1) a *p* value ≤ 0.01; (2) > 1.28-fold greater than the expression in other clusters; and (3) genes expressed in more than 25% of the cells in the target cluster.

Pathway enrichment analysis identified significantly enriched metabolic pathways or signal transduction pathways in DEGs compared with the whole-genome background. The *p* value was passed through false discovery rate (FDR) correction, taking an FDR ≤ 0.05 as the threshold. Pathways meeting these conditions were defined as significantly enriched pathways among the DEGs.

### Single-cell trajectories analysis

Single-cell trajectory was analysed using a matrix of cells and gene expression data with PAGA [71]. Monocle reduced the space down to one with two dimensions and ordered the cells (sigma = 0.001, lambda = NULL, param.gamma = 10, tol = 0.001). Once the cells were ordered, the trajectory was visualized in the reduced dimensional space.

### IF

The gonads from orange-spotted grouper were sampled and then embedded in optimal cutting temperature compound (Sakura, Japan). Fast-cooled tissue blocks were cut into serial sections (5 μm thick). IF analysis was performed using a modified method as previously described [28]. Briefly, the frozen gonadal sections were rehydrated in PBS for 30 min at room temperature. After antigen retrieval, the sections were subjected to hydrogen peroxide for 30 min, blocked with 5% BSA (Sigma, Germany) in PBS, and incubated with primary antibodies against Vasa, Piwil1, Gsdf, and PCNA (1:100 diluted with 2% BSA) overnight at 4 °C. After being washed several times with PBS, the sections were blocked with 5% goat serum in PBS for 1 h and incubated for 1 h at room temperature with a secondary HRP-conjugated anti-mouse/rabbit IgG antibody (Boster, China) at a dilution of 1:2000 with 2% goat serum in PBS. The sources of the antibodies are listed in Additional file 2: Table S4. Finally, the sections were observed under a light microscope (Leica, TCS-SP5, Germany).

### Generation of genetically modified SSC and HKE293T cells

SSC cells were derived from mature male testes and were passaged for more than 120 generations continuously. SSC cells were grown in Dulbecco’s modified Eagle’s medium/Ham’s F-12 (Advanced DMEM/F-12) (Gibco®, Waltham, MA, USA) supplemented with 5% foetal bovine serum (Gibco®) and 1% penicillin‒streptomycin (Gibco®, Waltham, MA, USA) at 28 °C and 5% CO_2_ [51]. HEK293T cells were preserved from our laboratory and were grown in Dulbecco’s modified Eagle’s medium (DMEM) (Gibco®, Waltham, MA, USA) supplemented with 10% foetal bovine serum (VivaCell, Shanghai, China) and 1% penicillin‒streptomycin (Gibco®, Waltham, MA, USA) at 37 °C and 5% CO_2_ [72]. The pEGFP-N3 vector was selected, and the full-length *piwil1* and *dmrt1* genes, which were cloned with specific restriction sites at both ends, were ligated to the vector. The vector was subsequently cotransfected into GPT cells or HEK293T cells with Lipofectamine 3000 (Invitrogen, Carlsbad, CA, USA) according to the manufacturer’s protocol. After a 48-h incubation posttransduction, the infection efficiency was assessed by observing GFP fluorescence under an inverted fluorescence microscope (Lecia, DMi8, Germany). Afterwards, the expression of *piwil1* and *dmrt1* was confirmed using RT‒qPCR. Finally, the cells that exhibited high infection efficiency and that overexpressed *piwil1* and *dmrt1* were selected for the detection of other genes. The primers used for the genes selected are listed in Additional file 2: Table S5.

### Statistical analysis

All the data are expressed as the mean values ± SEMs. Significant differences were checked by one-way analysis of variance (ANOVA) and Student’s *t* test, and a probability level < 0.05 (*P* < 0.05) was considered to indicate statistical significance. All the data were analysed using GraphPad Prism 5.0 (GraphPad Software, San Diego, CA).

## Supplementary Information

Additional file 1: Fig. S1-5. Fig. S1, Histology and genes expression changes of gonad during sex reversal induced by MT nine weeks. Fig. S2, Quality control of the eight samples. Fig. S3, UMAP plots of 14 marker genes in somatic cell clusters. Fig. S4, Cell numbers and UMAP plots of four subclusters from GSC in four gonadal stages. Fig. S5, UMAP plots of 9 marker genes in interstitial cell subclusters.

Additional file 2: Table S1-5. Table S1. The overview quality of scRNA-seq data. Table S2. The cell number and percentage of every cluster in scRNA-seq data. Table S3. The primers of selected genes by RT-qPCR during sex reversal. Table S4. Key reagent/resources in this paper. Table S5. The primers of selected genes by overexpression in SSC and HKE293T cells.

## Data Availability

In present study, scRNA-seq data were uploaded to NCBI database (https://www.ncbi.nlm.nih.gov/) under accession number PRJNA1277545 (http://www.ncbi.nlm.nih.gov/bioproject/PRJNA1277545/) [73].

## Abbreviations

scRNA-seq: Single-cell transcriptome sequencing
MT: 17α-methyltestosterone
GSC: Germ stem cell
GSD: Genotypic sex determination
ESD: Environmental sex determination
OSC: Oogonial stem cell
SSC: Spermatogonial stem cell
PGO: Primary-growth oocyte
PVO: Cortical-alveolus oocytes
SG: Spermatogonia
SC: Spermatocyte
(f): Natural female
(i): The intermediate stage of sex reversal
(im): MT induced male
(m): Natural male
UMI: Unique molecular identifier
UMAP: Uniform Manifold Approximation and Projection
DEG: Differential expressed gene
OG: Oogonia
lpSC: Late primary spermatocyte
pSC: Primary spermatocyte
ST: Spermatid
preOC: Pre-oocyte
OC: Oocyte
HEK-293T: Human embryonic kidney 293T
TUNEL: Terminal deoxynucleotidyl transferase dUTP nick end labelling
PCNA: Proliferating cell nuclear antigen
PAGA: Partition-based graph abstraction
FDR: False discovery rate
IF: Immunofluorescence
H&E: Hematoxylin-eosin
RT-qPCR: Real-time quantitative PCR
PCA: Principal component analysis
GEM: Gel Beads-In-Emulsion
DMEM: Dulbecco’s modified Eagle’s medium
ANOVA: One-way analysis of variance
