# Supplementary material for: Single-cell transcriptomics reveals the differential landscape of gonadal cells during MT-induced sex reversal in hermaphroditic protogynous orange-spotted grouper (Epinephelus coioides)
Source: BMC Biol. 2026 Jul 27;24:223. doi: 10.1186/s12915-026-02695-4 (PMC13700244; doi:10.1186/s12915-026-02695-4)
Supplement: Supplementary file 1 [file 12915_2026_2695_MOESM1_ESM.docx]

Additional file 1: Fig. S1 Histology and genes expression changes of gonad during sex reversal induced by MT nine weeks. (A) The morphology of female ovary without MT treatment. (B-F) The morphology of gonad after MT implantation 1 week, 3 weeks, 6 weeks, 7 weeks, and 9 weeks. Scale bar = 50 μm. (G-P) The expression of ten genes (*dnd*, *esg1*, *nanog*, *pou5f1a*, *dmrt1*, *npm1*, *h1foo*, *slbp2*, *ybx2a*, *zar1*) during 9 weeks after MT implantation. A and b indicate different level of significance.

Additional file 1: Fig. S2 Quality control of the eight samples. (A) The microscopy views of four different groups (f, i, im, and m). (B) Different UMI count of single-cell transcriptomic data in eight samples. (C) Different gene density of single-cell transcriptomic data in eight samples.

Additional file 1: Fig. S3 UMAP plots of 14 marker genes in somatic cell clusters. The bluer dots represent the higher expression.

Additional file 1: Fig. S4 Cell numbers and UMAP plots of four subclusters from GSC in four gonadal stages. (A) Cell numbers of four subclusters from GSC in four gonadal stages. The asterisk (*) represents the level of significance, and ns represents no significance. (B) UMAP plots of the transcripts level from four subclusters in GSC. (C-E) UMAP plots of three proliferative marker genes (*pcna*, *mki67*, *mcm4*) in GSC subclusters. The purplier dots represent the higher expression.

Additional file 1: Fig. S5 UMAP plots of 9 marker genes in interstitial cell subclusters. The bluer dots represent the higher expression.
