# Supplementary material for: Single-cell transcriptomics reveals the differential landscape of gonadal cells during MT-induced sex reversal in hermaphroditic protogynous orange-spotted grouper (Epinephelus coioides)
Source: BMC Biol. 2026 Jul 27;24:223. doi: 10.1186/s12915-026-02695-4 (PMC13700244; doi:10.1186/s12915-026-02695-4)
Supplement: Supplementary file 2 [file 12915_2026_2695_MOESM2_ESM.docx]

Additional file 2: Table S1 The overview quality of scRNA-seq data.

| **Sample** | **Cell number after filtration** | **UMI** | **UMI/cell** | **Mitochondria UMI** | **Sample saturation** |
| --- | --- | --- | --- | --- | --- |
| F-1 | 5726 | 43564720 | 7608.23 | 0.07% | 44.20% |
| F-2 | 4851 | 27761285 | 5722.8 | 0.53% | 79.30% |
| I-1 | 12439 | 40347361 | 3243.62 | 0.51% | 70.40% |
| I-2 | 8315 | 30004006 | 3608.42 | 0.29% | 56.90% |
| IM-1 | 8867 | 32554263 | 3671.4 | 0.40% | 78.40% |
| IM-2 | 7530 | 41496011 | 5510.76 | 0.45% | 58.80% |
| M-1 | 7534 | 27391305 | 3635.69 | 0.68% | 81.70% |
| M-2 | 5749 | 21050076 | 3661.52 | 0.67% | 87.80% |

Additional file 2: Table S2 The cell number and percentage of every cluster in scRNA-seq data.

| **Cluster** | **(f)** | **(i)** | **(im)** | **(m)** | **Total** |
| --- | --- | --- | --- | --- | --- |
| 0 | 63 (0.62%) | 3109 (13.98%) | 1865 (11.09%) | 2278 (16.83%) | 7315 |
| 1 | 109 (1.08%) | 2922 (13.14%) | 1300 (7.73%) | 2886 (21.32%) | 7217 |
| 2 | 1742 (17.18%) | 2070 (9.31%) | 3281 (19.51%) | 99 (0.73%) | 7192 |
| 3 | 4017 (39.63%) | 1964 (8.83%) | 545 (3.24%) | 160 (1.18%) | 6686 |
| 4 | 12 (0.12%) | 2620 (11.78%) | 1533 (9.11%) | 1801 (13.3%) | 5966 |
| 5 | 19 (0.19%) | 1554 (6.99%) | 1393 (8.28%) | 2396 (17.7%) | 5362 |
| 6 | 11 (0.11%) | 2285 (10.27%) | 1194 (7.1%) | 1176 (8.69%) | 4666 |
| 7 | 1165 (11.49%) | 1028 (4.62%) | 2147 (12.76%) | 40 (0.3%) | 4380 |
| 8 | 24 (0.24%) | 1134 (5.1%) | 563 (3.35%) | 1082 (7.99%) | 2803 |
| 9 | 901 (8.89%) | 271 (1.22%) | 408 (2.43%) | 188 (1.39%) | 1768 |
| 10 | 659 (6.5%) | 271 (1.22%) | 766 (4.55%) | 51 (0.38%) | 1747 |
| 11 | 17 (0.17%) | 484 (2.18%) | 376 (2.24%) | 574 (4.24%) | 1451 |
| 12 | 397 (3.92%) | 470 (2.11%) | 191 (1.14%) | 305 (2.25%) | 1363 |
| 13 | 174 (1.72%) | 407 (1.83%) | 159 (0.95%) | 232 (1.71%) | 972 |
| 14 | 98 (0.97%) | 379 (1.7%) | 127 (0.76%) | 194 (1.43%) | 798 |
| 15 | 168 (1.66%) | 320 (1.44%) | 242 (1.44%) | 2 (0.01%) | 732 |
| 16 | 274 (2.7%) | 391 (1.76%) | 38 (0.23%) | 27 (0.2%) | 730 |
| 17 | 146 (1.44%) | 185 (0.83%) | 210 (1.25%) | 38 (0.28%) | 579 |
| 18 | 4 (0.04%) | 232 (1.04%) | 256 (1.52%) | 3 (0.02%) | 495 |
| 19 | 103 (1.02%) | 49 (0.22%) | 182 (1.08%) | 4 (0.03%) | 338 |
| 20 | 34 (0.34%) | 94 (0.42%) | 45 (0.27%) | 2 (0.01%) | 175 |
| Total | 10137 | 22239 | 16821 | 13538 | 62735 |

Additional file 2: Table S3 The primers of selected genes by RT-qPCR during sex reversal.

| Gene | Primer Sequences |
| --- | --- |
| dnd-F | TGCGAGGCGAGTGGTTT |
| dnd-R | CGTTTCCCGGCAAGATC |
| esg1-F | ACGGCAAGAAGAGGAAAGC |
| esg1-R | TCCCTGGACGAGTAGGACTG |
| nanog-F | GGAGGCACCAGAAAGACACCA |
| nanog-R | CTGAGTCAGGGCTTCTCCTTGATA |
| pou5f1-F | TGTCCCAAGCCCAACACCCA |
| pou5f1-R | CCAGGCGCTTTCCTTTCTGTC |
| dmrt1-F | CTGTAGAAACCACGGCTATGT |
| dmrt1-R | GGATCGTCTCTCCACAGTAAAC |
| npm1-F | GGTCCAATCCACCTCAGTG |
| npm1-R | CATCGTCCTCGTCATCCTC |
| h1foo-F | TCACCCATCCACAGCCATTA |
| h1foo-R | CGTGCCAGTCTCGATTCCTT |
| slbp2-F | ACATGACCCAGTGGAGCAA |
| slbp2-R | TCACAGCCGACAGAGGAGAC |
| ybx2a-F | CCCAGGCGGTGTTCCAGTCA |
| ybx2a-R | CCCTCCTGCCGTGGTCTTCG |
| zar1-F | GCCTTCAACAACCACCAGC |
| zar1-R | TGCACCGATGCGTCTTTC |

Additional file 2: Table S4 Key reagent/resources in this paper.

| Reagent/Resource | Source | Identifier |
| --- | --- | --- |
| Antibodies | | |
| Rabbit anti-Vasa | Dia-an | 2008 |
| Rabbit anti-Piwi | this paper | N/A |
| Rabbit anti-Gsdf | this paper | N/A |
| Mouse anti-Pcna | Proteintech | 10205-2-AP |
| Goat anti-rabbit IgG secondary antibody, HRP | Boster | BA1054 |
| Chemicals, Peptides and Recombinant Proteins | | |
| MS222 | Sigma | W246719-1KG-K |
| Hematoxylin | Sigma | H3136-25G |
| Eosin | Sigma | 230251-25G |
| Paraffin | Lecia | 39601006 |
| 1×Hank's Balanced Salt Solution | HyClone | SH30588.01 |
| Collagenase IV | Sigma | C1889-50MG |
| Anti-Digoxigenin-AP Fab fragments | Roche | 11093274910 |
| Anti-Digoxigenin-POD, Fab fragments | Roche | 11207733910 |
| DAPI | Sigma | D9542 |
| Optimal cutting temperature compound | Sakura | 4583 |
| Hydrogen peroxide | Thermofish | C20246 |
| BSA | Sigma | A3311 |
| Goat serum | Sigma | G9023 |
| Deposited Data | | |
| Single cell RNA-seq data | NCBI database | PRJNA1277545(SAMN49112660-SAMN49112667) |

Additional file 2: Table S5 The primers of selected genes by overexpression in SSC and HKE293T cells.

| Gene | Primer Sequences |
| --- | --- |
| dmrt1-F | GGCTATGTGTCTCCTCTGAA |
| dmrt1-R | ATTCTTCACCATCACCTCAG |
| dazl-F | TGGGTAGGGGTATGGCACAA |
| dazl-R | TGGGCGTTCTGTACATGGTT |
| sycp3-F | AGTGGTGCAGAACCAGAAACTG |
| sycp3-R | TGTTGCGTGTCCATGAGGAT |
| piwil1-F | TAGGCGATACCCCACAAGGA |
| piwil1-R | CTCAGTTGCTGGGAAGAGCA |
| sox2-F | GGAGGAAGATGGCGCAAGAA |
| sox2-R | TCTCTTCTCGCTCTCCGACA |
| dnmt3b-F | TCAACATCTGCCGCTTCCTT |
| dnmt3b-R | GTCGTTCTGGGACGCTGTAA |
